# Fibroblast growth factor 23 and fibroblast growth factor receptor 4 promote cardiac metabolic remodeling in chronic kidney disease

**DOI:** 10.1016/j.kint.2025.01.024

**Published:** 2025-02-07

**Authors:** Michaela A.A. Fuchs, Emily J. Burke, Nejla Latic, Susan L. Murray, Hanjun Li, Matthew A. Sparks, Dennis Abraham, Hengtao Zhang, Paul Rosenberg, Umber Saleem, Arne Hansen, Sara E. Miller, Davis Ferreira, Sonja Hänzelmann, Fabian Hausmann, Tobias Huber, Reinhold G. Erben, Kelsey Fisher-Wellman, Nenad Bursac, Myles Wolf, Alexander Grabner

**Affiliations:** 1 Division of Nephrology, Department of Medicine, Duke University School of Medicine, Durham, North Carolina, USA; 2 Department of Biomedical Sciences, University of Veterinary Medicine, Vienna, Austria; 3 Department of Biomedical Engineering, Duke University, Durham, North Carolina, USA; 4 Division of Cardiology, Department of Medicine, Duke University School of Medicine, Durham, North Carolina, USA; 5 Department of Experimental Pharmacology and Toxicology, University Medical Center Hamburg-Eppendorf, Hamburg, Germany; 6 German Center for Heart Research (DZHK), Partner Site Hamburg/Lübeck/Kiel, Germany; 7 Department of Pathology, Duke University, Durham, North Carolina, USA; 8 Institute of Medical Systems Biology, University Medical Center Hamburg-Eppendorf, Hamburg, Germany; 9 Center for Biomedical AI, University Medical Center Hamburg-Eppendorf, Hamburg, Germany; 10 Division of Nephrology, Department of Medicine, University Medical Center Hamburg-Eppendorf, Hamburg, Germany; 11 Hamburg Center for Kidney Health (HCKH), University Medical Center Hamburg-Eppendorf, Hamburg, Germany; 12 Ludwig Boltzmann Institute of Osteology, Hanusch Hospital, Vienna, Austria; 13 Department of Physiology, East Carolina Diabetes and Obesity Institute, East Carolina University, Greenville, North Carolina, USA; 14 Department of Physiology, Brody School of Medicine, East Carolina University, Greenville, North Carolina, USA; 15 Department of Physiology, UNC Lineberger Comprehensive Cancer Center, University of North Carolina at Chapel Hill School of Medicine, Chapel Hill, North Carolina, USA; 16 Duke Regeneration Center, Duke University, Durham, North Carolina, USA; 17 Duke Clinical Research Institute, Duke University, Durham, North Carolina, USA; 18 Joan and Sanford I. Weill Department of Medicine, Weill Cornell Medicine, New York, New York, USA

**Keywords:** CKD, FGF23, FGFR4, heart failure, metabolic remodeling, mitochondrial dysfunction

## Abstract

Chronic kidney disease (CKD) is a global health epidemic that greatly increases mortality due to cardiovascular disease. Left ventricular hypertrophy (LVH) is an important mechanism of cardiac injury in CKD. High serum levels of fibroblast growth factor (FGF) 23 in patients with CKD may contribute mechanistically to the pathogenesis of LVH by activating FGF receptor (FGFR) 4 signaling in cardiac myocytes. Mitochondrial dysfunction and cardiac metabolic remodeling are early features of cardiac injury that predate development of hypertrophy, but these mechanisms have been insufficiently studied in models of CKD. We found in wild-type mice with CKD induced by adenine diet, that morphological changes occurred in mitochondrial structure and cardiac mitochondrial and that metabolic dysfunction preceded the development of LVH. In bioengineered cardio-bundles and neonatal rat ventricular myocytes grown *in vitro*, FGF23-mediated activation of FGFR4 caused mitochondrial pathology, characterized by increased bioenergetic stress and increased glycolysis that preceded the development of cellular hypertrophy. The cardiac metabolic changes and associated mitochondrial alterations in mice with CKD were prevented by global and cardiac-specific deletion of FGFR4. Our findings indicate that metabolic remodeling and mitochondrial dysfunction are early cardiac complications of CKD that precede structural remodeling of the heart. Mechanistically, FGF23-mediated activation of FGFR4 causes mitochondrial dysfunction, suggesting that early pharmacologic inhibition of FGFR4 might serve as novel therapeutic intervention to prevent development of LVH and heart failure in patients with CKD.

Chronic kidney disease (CKD) is a major health epidemic that affects millions of people worldwide and increases risks of cardiovascular disease and mortality.^1–3^ Left ventricular hypertrophy (LVH) causes heart failure with preserved ejection fraction (HFpEF), which affects most patients with CKD.^4^ Among patients with CKD, alteration in mineral homeostasis is an additional unique factor in the complex multifactorial pathogenesis of HFpEF.^5–7^

Fibroblast growth factor (FGF) 23 regulates calcium and phosphate homeostasis.^8^ In CKD, FGF23 levels progressively increase as kidney function declines. Elevated FGF23 helps maintain normal serum phosphate in CKD,^9^ but higher FGF23 is dose dependently associated with increased risks of LVH, heart failure, and mortality in patients with CKD.^7,10–12^ Mechanistically, FGF23 induces LVH in rodents by activating FGF receptor (FGFR) 4 and the phospholipase Cγ–calcineurin–nuclear factor of activated T cells signaling pathway,^10,13–16^ which is a potent inducer of structural cardiac remodeling.^17^ Human genetic data support the link between FGF23 and development of LVH and heart failure, particularly in patients who are genetically predisposed to developing CKD.^18^

Mounting evidence suggests that metabolic remodeling and mitochondrial dysfunction are upstream mechanisms of LVH that precede other pathologic alterations.^19^ Metabolic inefficiency and loss of coordinated anabolic activity have emerged as proximal causes of cardiac structural remodeling.^20,21^ Despite the high rates of structural cardiac remodeling in CKD,^6^ the specific role of cardiac metabolism in heart failure associated with CKD remains largely underexplored.^22^ Furthermore, no studies investigated whether FGF23-FGFR4 activation contributes to cardiac metabolic remodeling and mitochondrial dysfunction.^23^ Using bioengineered cardiobundles, cultured cardiomyocytes, and multiple rodent models of CKD, we tested the hypotheses that CKD induces cardiac mitochondrial dysfunction and metabolic remodeling; that these changes predate the development of structural cardiac remodeling; and that FGF23-FGFR4 activation is a molecular mechanism of these effects.

## METHODS

### Antibodies, recombinant proteins, and heparin

Carrier-free recombinant mouse FGF23 (catalog number 2629-FG025/CF, R&D Systems) was used at 25 and 100 ng/ml. The isoform-specific FGFR4 small-molecule inhibitor BLU9931 (catalog number S7819, Selleck Chemicals) was used at 10 ng/ml. Heparin solution (McKesson Corporation) was used at 0.2 United States Pharmacopeia units/ml. Primary antibodies include sarcomeric a-actinin (catalog number EA-53, Sigma-Aldrich). Secondary antibody is Cy3-conjugated goat–anti-mouse (catalog number 115165166, Jackson ImmunoResearch Laboratories).

### Isolation and cultivation of neonatal rat ventricular myocytes

Neonatal rat ventricular myocytes (NRVMs) were isolated from Sprague-Dawley rat pups (postnatal day 0–3) using a commercially available kit (catalog number LK003300, Worthington), as described previously and in the Supplementary Methods.^24^

### Fabrication of bioengineered cardiobundles

Bioengineered cardiobundles were generated as described previously and in the Supplementary Methods.^25^ In selected studies, FGF23 (25 ng/ml) and BLU9931 (10 ng/ml) were added to culture medium on day 7 and replenished with each medium change during the following 7 days.

### Measurement of contractile force and action potential propagation

Isometric contractile forces were measured as described previously.^25^ In brief, cardiobundles were immersed in Tyrode solution containing 1.8 mM CaCl_2_ and connected to a force transducer. Contractions were elicited by electric field stimulus from parallel platinum electrodes. Optical mapping of action potentials was performed as described previously.^26^ Cardiobundles were stained with a transmembrane voltage-sensitive dye (di-4-ANEPPS [6-[2-(N,N-Dibutylamino)naphthyl]ethenyl-4′-pyridinium propanesulfonate]) and paced at different rates by suprathreshold point stimulus to map propagation of action potentials.

### Immunofluorescence and morphometry of cultured myocytes and cardiobundles

Hypertrophic growth of isolated NRVMs was analyzed on laminin-coated glass coverslips after 48 hours of treatment, as reported previously and as described in the Supplementary Methods.^13,25,27^

### Live-cell metabolic analysis

Mitochondrial oxygen consumption rate and glycolytic rate were determined in NRVMs using the Seahorse XF Mito Stress Test Kit and the Seahorse XF Glycolytic Rate Assay Kit, according to manufacturer’s protocols. All live-cell metabolic assays were performed in collaboration with Duke’s Cardiovascular Physiology Core using the metabolic flux analyzer Agilent Seahorse XF96 (Agilent Technologies); see Supplementary Methods for details and Supplementary References.

### Differentiation of human induced pluripotent stem cell to engineered heart tissue and analysis of contraction force and frequency

Human induced pluripotent stem cells (cell line: ERC001) were differentiated into cardiomyocytes and then used for the generation of engineered heart tissue, as described previously.^28–30^ The contraction force of engineered heart tissues was recorded using automated video-optical analysis, as described previously.^29,30^ See Supplementary Methods for details.

### RNA isolation and quantification

Total RNA was extracted from hearts and cultured cardiomyocytes using a RNeasy Plus Mini Kit (Qiagen) following the manufacturer’s instructions. A total of 0.5 to 2 μg of RNA was reverse transcribed to cDNA using Applied Biosystems High-Capacity cDNA Reverse Transcription Kit (catalog number 4368813, ThermoFisher). Quantitative real-time polymerase chain reaction was performed in duplicate with the SSoAdvanced Universal Probe Supermix (Bio-Rad) and sequence-specific TaqMan probes (ThermoFisher), as indicated in Table 1, on a Quantstudio 3 (Applied Biosystems, ThermoFisher). Gene expression was normalized to expression levels of housekeeping genes β2-microglobulin (for *in vitro* studies) or 18S rRNA (for *in vivo* studies). Results were evaluated using the 2^−ΔΔCt^ method and expressed as mean ± SEM.

### Mice

Constitutive FGF receptor 4 null mice (FGFR4^−/−^),^31^ constitutive collagen type IV alpha 3 (Col4a3)^−/−^ mice,^32^ and constitutive FGFR4 knock-in mice (FGFR4-Arg385)^33^ were used. Mice with inducible cardiomyocyte-specific deletion of FGFR4 (alpha-myosin heavy chain [MHC]^MerCreMer^–FGFR4^flox^) were generated by crossing α-MHC^MerCreMer^ mice^34^ with FGFR4 floxed mice.^35^ All mice were maintained on a C57Bl/6 background. Cre recombination was induced by tamoxifen injections (30 mg/kg body weight, i.p., every 48 hours for a total of 3 injections). Dietary interventions were started 10 days after the last tamoxifen injection. Male and female mice were used in the distribution indicated in the Supplementary Methods.

### Adenine model of CKD

As described previously, CKD was induced by feeding 12- to 16-week-old mice an adenine containing diet (0.15%, TD.170304, to 0.2% adenine, TD.140290; control diet, TD.170303, Envigo).^36^ All mice were put on control diet for 1 week before study start, and then mice were randomized to receive either adenine diet or control diet.

### Noninvasive assessment of kidney function

Glomerular filtration rate was determined noninvasively in mice using non-invasive clearance kidney devices, as described previously and in the Supplementary Methods.^37,38^

### Noninvasive and invasive assessment of cardiac function

All echocardiographic analyses were performed by Duke’s Cardiovascular Physiology Core using a Vevo 3100 imaging system (FUJIFILM VisualSonics); see Supplementary Methods for details.

### Serum chemistry

At study end, blood was collected from mice at the time of termination via cardiac puncture, transferred into Microvette heparin plasma tubes (Sarstedt), and centrifuged at 21,000*g* for 10 minutes. Plasma supernatants were collected and stored at −80 °C. Blood urea nitrogen, phosphate, and hemoglobin were measured at the University of North Carolina Animal Histopathology and Lab Medicine Core with an Alfa Wassermann Vet Axcel Chemistry Analyzer (Alfa Wassermann Diagnostic Technologies, LLC). Intact and C-terminal FGF23 levels and parathyroid hormone 1-84 were determined by enzyme-linked immunosorbent assay (QuidelOrtho), according to the manufacturer’s protocol.

### Mitochondrial respiration

Mitochondrial isolation from frozen mouse hearts was performed as previously described and in the Supplementary Methods.^39^ High-resolution oxygen consumption rate was assessed via the Oroboros Oxygraph-2K (Oroboros Instruments), as previously described,^39^ with minor adjustments (see Supplementary Methods for details).

### RNA sequencing

RNA sequencing was performed in collaboration with Duke’s Center for Genomic and Computational Biology Core Facility. See Supplementary Methods for further details.

### Proteomics

Proteomics of isolated cardiac mitochondria was performed in collaboration with Duke’s Proteomics and Metabolomics Core Facility. See Supplementary Methods for detailed description and references.

### Metabolomics

Metabolomic measurements were performed at the Metabolomics Core Laboratory at Duke Molecular Physiology Institute. See Supplementary Methods for detailed description and references.

### Transmission electron microscopy

Transmission electron microscopy (TEM) was performed by Duke’s Center for Electron Microscopy and Nanoscale Technology. See Supplementary Methods for detailed description.

### Semiquantitative analysis of mitochondrial morphology

TEM of cardiac tissue and selection of view fields was performed by Dr. Davis Ferreira of the Duke Center for Electron Microscopy and Nanoscale Technology in a blinded manner. Analysis of mitochondrial morphology was independently performed by 2 investigators (MAAF and EJB) in a blinded manner using ImageJ.^40^ See Supplementary Methods for details.

### Study approval

All animal protocols and experimental procedures were approved by the Institutional Animal Care and Use Committees at Duke University. All animals were maintained in a ventilated rodent-housing system with temperature-controlled environments (22 °C–23 °C) with a 12-hour light/dark cycle and allowed *ad libitum* access to food and water. All protocols adhered to the *Guide for Care and Use of Laboratory Animals* to minimize pain and suffering. Experiments were designed and conducted in accordance with the ARRIVE (Animal Research: Reporting of *In Vivo* Experiments) guidelines.^41^ No animals were excluded from analysis.

### Statistical analysis

All data are presented as means ± SEM. *P* < 0.05 was considered statistically significant. All data were analyzed using GraphPad Prism9 (Graphpad Software), followed by Student *t*-tests or, when appropriate, by 2-way analysis of variance with Šidák correction for multiple comparisons. See Supplementary Methods for more details.

### Graphical design

Schematics of the graphical abstract were designed using the BioRender Software (Fuchs M [2024]; https://BioRender.com/x67j640).

## RESULTS

### CKD alters cardiac mitochondrial structure and function before the onset of LVH

**Figure 1 |:**
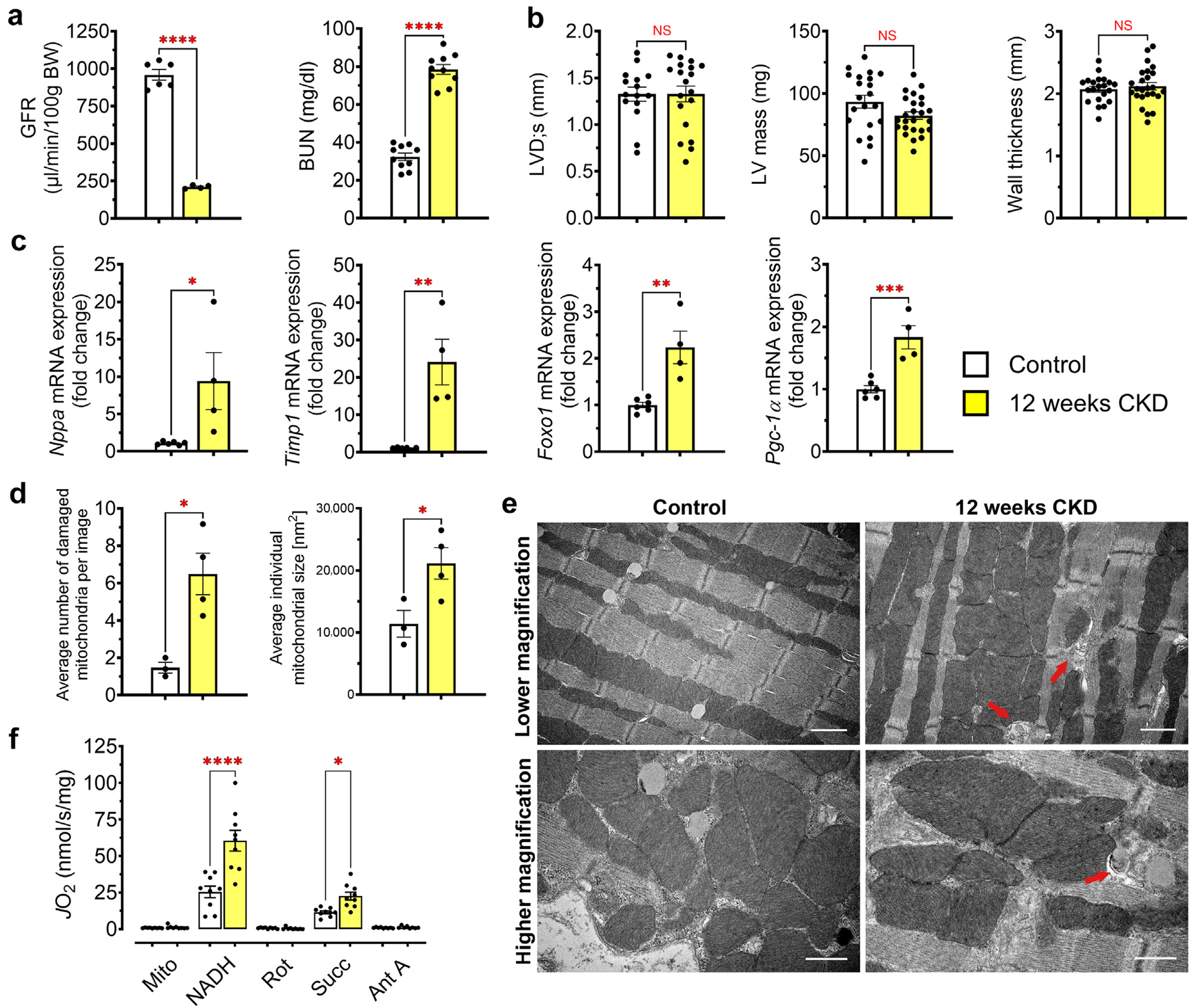
Cardiac function, remodeling, and changes to cardiac mitochondria in chronic kidney disease (CKD). Renal and cardiac function of mice was evaluated 12 weeks after starting adenine-containing diet to induce CKD or control diet. (**a**) Measurement of glomerular filtration rate (GFR) and blood urea nitrogen (BUN) indicated significant kidney damage in mice fed adenine diet when compared with mice fed control diet. (**b**) Cardiac functional parameters of mice after 12 weeks of CKD did not indicate manifestation of impaired function or significant structural remodeling, as demonstrated by left ventricular end-systolic diameter (LVD;s), left ventricular (LV) mass, and wall thickness. (**c**) Expression levels of remodeling parameters *Nppa, Timp1, Foxo1*, and *Pgc-1α* indicate that hypertrophic and fibrotic remodeling had been initiated at 12 weeks of CKD. (**d**) Semiquantitative evaluation of mitochondria by electron microscopy revealed significant changes in cardiac mitochondrial morphology of mice with CKD. Heart tissue of mice with CKD showed a significantly increased number of damaged mitochondria per field of view and increased average mitochondrial size, further indicating mitochondrial dysfunction. (**e**) Representative images of electron microscopy demonstrate swelling and misalignment of mitochondria in CKD hearts. Damaged mitochondria are indicated by red arrows. Bar = 1 μm for lower-magnification images and 600 nm for higher-magnification images. (**f**) Mitochondrial (Mito) respiration after 12 weeks of adenine diet showed that respiration through complex I and II was significantly increased before structural cardiac remodeling was detectable. (**a–c**) Bar graphs represent mean ± SEM with individual values included in the graph, n ≥ 4 male mice for all experiments. *P < 0.05, **P < 0.005, ***P < 0.0005, ****P < 0.0001. Quantification of transmission electron microscopy images was performed on tissue of male and female mice. Ant A, antimycin A; *J*O_2_, oxygen consumption rate; NADH, reduced nicotinamide adenine dinucleotide; NS, not significant; Rot, rotenone; Succ, succinate. To optimize viewing of this image, please see the online version of this article at www.kidney-international.org.

We analyzed cardiac structure and function in wild-type mice fed 0.2% adenine diet to induce CKD.^36^ Adenine-induced CKD caused LVH after 16 weeks, as indicated by increased left ventricular (LV) mass index, increased posterior wall thickness, and reduced systolic LV diameter. LV function, marked by fractional shortening, was unchanged (Supplementary Figure S1A). To investigate cardiac metabolism and remodeling in the setting of CKD before the onset of LVH, we analyzed mice after 12 weeks of adenine diet. At this stage, reduced glomerular filtration rate and increased blood urea nitrogen confirmed severe kidney damage, but cardiac structural remodeling was not yet evident in male or female mice (Figure 1a and b and Supplementary Figure S1B). Cardiac expression of the hypertrophy and fibrosis markers *Nppa* and *Timp1* mRNA was increased after 12 weeks of adenine diet, indicating that molecular processes of hypertrophic and fibrotic remodeling had already begun (Figure 1c). Interestingly, expression of the transcription factors *Pgc-1α* and *Foxo1*, which are key regulators of myocardial metabolism and cardiac mitochondrial function, was also significantly increased in CKD versus controls after 12 weeks of adenine feeding (Figure 1c). At this time point, TEM revealed grossly normal myofibrillar structures in the CKD myocardium, but mitochondria were misaligned, swollen, and larger than in control animals; cristae appeared more disorganized, and the number of damaged mitochondria was significantly increased, suggesting mitochondrial dysfunction (Figure 1d and e and Supplementary Figure S1C). Previous studies indicated sex differences in the adenine model.^42,43^ (For the purpose of this study, sex is definded as female [XX chromosomes] and male [XY chromosomes].) Analysis of female mice that had undergone 12 weeks of adenine feeding showed similar changes in functional and mitochondrial parameters as males with CKD (Supplementary Figure S1B and C).

To characterize myocardial mitochondrial function, we assessed respiratory capacity across the electron transport system. Respiration by respiratory chain complex I (reduced nicotinamide adenine dinucleotide supported) and complex II (succinate supported) was significantly increased in CKD hearts versus controls, suggesting that changes in cardiac mitochondrial function precede hypertrophic and fibrotic structural remodeling (Figure 1f).

**Figure 2 |:**
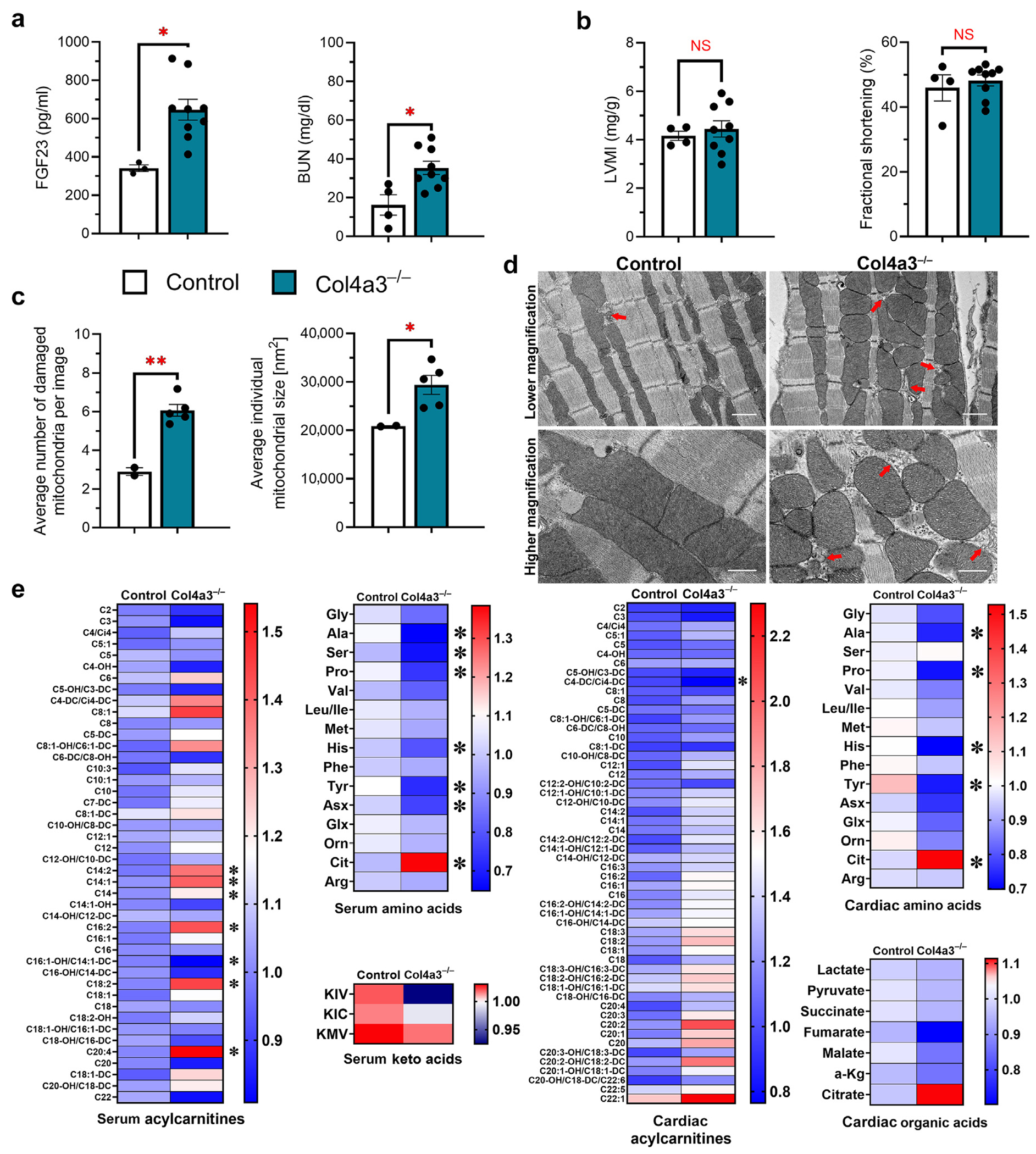
Renal function, cardiac characteristics, mitochondrial changes, and metabolome of collagen type IV alpha 3 (Col4a3)^−/−^ mice. Col4a3^−/−^ mice were evaluated for their renal, cardiac, and mitochondrial characteristics at 16 weeks. (**a**) Serum levels of intact fibroblast growth factor (FGF) 23 and blood urea nitrogen (BUN) indicate significant kidney damage at this time point, whereas (**b**) cardiac parameters determined by echocardiography did not yet show significant signs of cardiac hypertrophy or functional impairment. (**c**) Analysis of cardiac mitochondria revealed an increased number of damaged mitochondria and increased size of mitochondria in Col4a3^−/−^ mice compared with control animals without kidney damage. (**d**) Micrographs demonstrate swelling and misalignment of mitochondria in Col4a3^−/−^ hearts, similar to the morphologic changes observed in mice with adenine-induced chronic kidney disease; damaged mitochondria are indicated by red arrows. Bar = 1 μm for lower-magnification images and 600 nm for higher-magnification images. (**e**) Metabolomic analysis of serum and cardiac tissue of Col4a3^−/−^ mice revealed significant changes in serum acylcarnitines and amino acids, with a strong downward trend in keto acids. Changes to cardiac acylcarnitines in Col4a3^−/−^ mice trend toward increases predominantly in longer-chain acylcarnitines. Cardiac alanine and histidine were significantly downregulated and citrulline was upregulated, and other cardiac amino acids showed similar downward trends. Citrate showed a strong trend for upregulation in Col4a3^−/−^ mice, but did not reach significance. (**a–c**) Bar graphs represent mean ± SEM and individual values included in the graph, n ≥ 3 male mice for analysis of renal and cardiac parameters. For metabolomic analysis, 4 control animals and 6 Col4a3^−/−^ mice were evaluated. Quantification of transmission electron microscopy images was performed on tissue of male and female mice. *P < 0.05, **P < 0.005. KIC, ketoisocaproate; KIV, α-ketoisovalerate; KMV, ketomethylvalerate; LVMI, left ventricular mass index; NS, not significant. To optimize viewing of this image, please see the online version of this article at www.kidney-international.org.

To validate these findings in an alternative model of CKD, independent of potential confounding effects of adenine,^44^ we used a mouse model of human Alport disease (Col4a3^−/−^ mice).^32,45^ Because disease progression in Col4a3^−/−^ models is strain dependent, we selected slower-progressing C57BL6J/Col4a3^−/−^ mice that are known to eventually develop LVH.^45,46^ By 20 weeks of age, Col4a3^−/−^ mice exhibited LVH and changes in mitochondrial morphology (Supplementary Figure S2). At 16 weeks of age, Col4a3^−/−^ mice exhibited significantly elevated levels of blood urea nitrogen and FGF23 (Figure 2a), but showed no signs of structural cardiac remodeling (Figure 2b). TEM revealed increased numbers of damaged mitochondria and increased size of mitochondria in Col4a3^−/−^ versus control mice, paralleling our findings in the adenine model (Figure 2c and d). Mitochondria of Col4a3^−/−^ mice also appeared swollen, with more pronounced and disorganized cristae (Figure 2d). As in wild-type mice fed adenine, there was no sex difference in the cardiac phenotype of Col4a3^−/−^ mice (data not shown). Taken together, these results indicate that cardiac mitochondrial dysfunction precedes detectable structural changes in the hearts of male and female mice across different models of CKD.

### CKD changes the cardiac mitoproteome and metabolome

**Figure 3 |:**
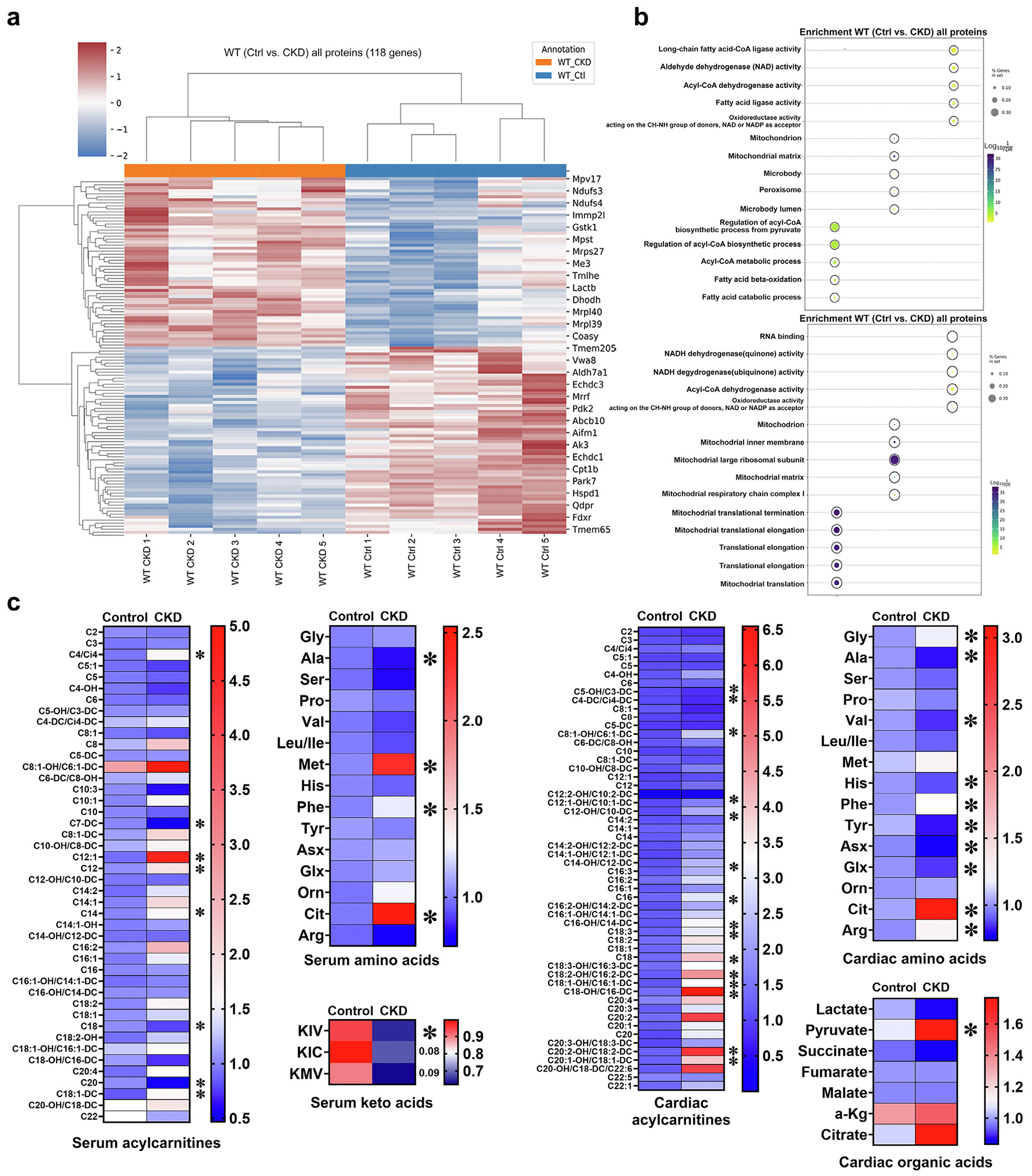
Changes to mitochondrial proteome and cardiac metabolome in chronic kidney disease (CKD). (**a,b**) CKD leads to significant changes in the mitochondrial proteome of wild-type (WT) mice after 12 weeks of adenine diet, before structural remodeling becomes detectable. (**a**) A total of 118 mitochondrial genes were significantly regulated in the mitoproteome of mice with CKD compared with controls (Ctrls). (**b**) Kyoto Encyclopedia of Genes and Genomes pathway analysis showed that downregulated proteins represented fatty acid metabolism and amino acid degradation (top). The upregulated genes were enriched in ribosomal processes and translation (bottom). (**c**) Analysis of cardiac and serum metabolome showed significant changes in serum acylcarnitines, amino acids, and keto acids (left). Among upregulated serum amino acids were phenylalanine and citrulline. These were also upregulated in cardiac tissue (right). Here, more amino acids like alanine and histidine were significantly downregulated, and leucine and isoleucine showed a downward trend (*P* = 0.07). In contrast to serum, more cardiac acylcarnitines, predominantly longer-chain acylcarnitines, were significantly upregulated. For organic acids from cardiac tissue of CKD mice only, pyruvate reached significance but lactate and citrate showed trends (*P* = 0.07). For proteomic analysis, n = 5 for each group; for serum and cardiac metabolomics, n ≥ 4 for each group. *P < 0.05. KIC, ketoisocaproate; KIV, α-ketoisovalerate; KMV, ketomethylvalerate; NAD, nicotinamide adenine dinucleotide; NADH, reduced nicotinamide adenine dinucleotide.

Next, we isolated mitochondria from the hearts of mice with CKD induced by 12 weeks of adenine diet to assess the cardiac mitoproteome using tandem mass spectrometry (Figure 3a). Using a previously reported mitochondrial enrichment factor,^47^ we achieved mitochondrial enrichment of ≈75% (data not shown). Of the 781 mitochondrial genes identified across CKD and control mice, 56 were upregulated and 62 were downregulated in CKD versus control hearts (Figure 3a). Downregulated proteins were significantly enriched in 6 different Kyoto Encyclopedia of Genes and Genomes pathways: fatty acid oxidation, acetyl-CoA metabolism, regulation of biosynthetic processes from pyruvate and reduced nicotinamide adenine dinucleotide phosphate, and antioxidant activity (Figure 3b, top). Upregulated proteins were involved in mitochondrial ribosomes and translation (Figure 3b, bottom). These results suggest that CKD induces functional changes in cardiac mitochondria.

To assess the cardiac metabolome in CKD, we performed targeted liquid chromatography–mass spectrometry analysis of serum and heart tissue (Figure 3c). Several medium- and long-chain acylcarnitines (MLACs) were significantly altered in CKD (Figure 3c). In serum, MLACs were mostly upregulated, whereas in cardiac tissue, some MLACs were upregulated and others downregulated when compared with controls. Several amino acids, including arginine, phenylalanine, and citrulline, were increased in hearts and serum of CKD mice, whereas cardiac concentrations of branched-chain amino acids, including valine and leucine/isoleucine, trended lower. In line, serum levels of branched-chain keto acids were also significantly reduced in CKD. Significant changes were detected in cardiac organic acids and tricarboxylic acid cycle intermediates. Pyruvate was significantly higher, whereas lactate trended lower in CKD versus controls (Figure 3c). Tricarboxylic acid cycle intermediates, including citrate, also trended higher in CKD hearts (Figure 3c). Taken together, these data suggest that CKD alters fatty acid, amino acid, and glucose metabolism in the heart.

Consistent with observations from the adenine model of CKD, the cardiac metabolome of 16-week-old Col4a3^−/−^ mice displayed comparable alterations. Notably, keto acids showed a pronounced downward trend, whereas longer-chain cardiac acylcarnitines exhibited an upward trend (Figure 2e). Alanine and histidine levels were significantly reduced, whereas citrulline was upregulated, closely mirroring the patterns observed in adenine-fed mice. Additionally, other branched-chain amino acids demonstrated a tendency toward lower levels, and citrate exhibited an upward trend. In summary, Col4a3^−/−^ mice and mice with CKD due to adenine share common alterations in the cardiac metabolome that precede the onset of structural cardiac remodeling.

### FGF23-FGFR4 induce hypertrophic growth of bioengineered cardiobundles

**Figure 4 |:**
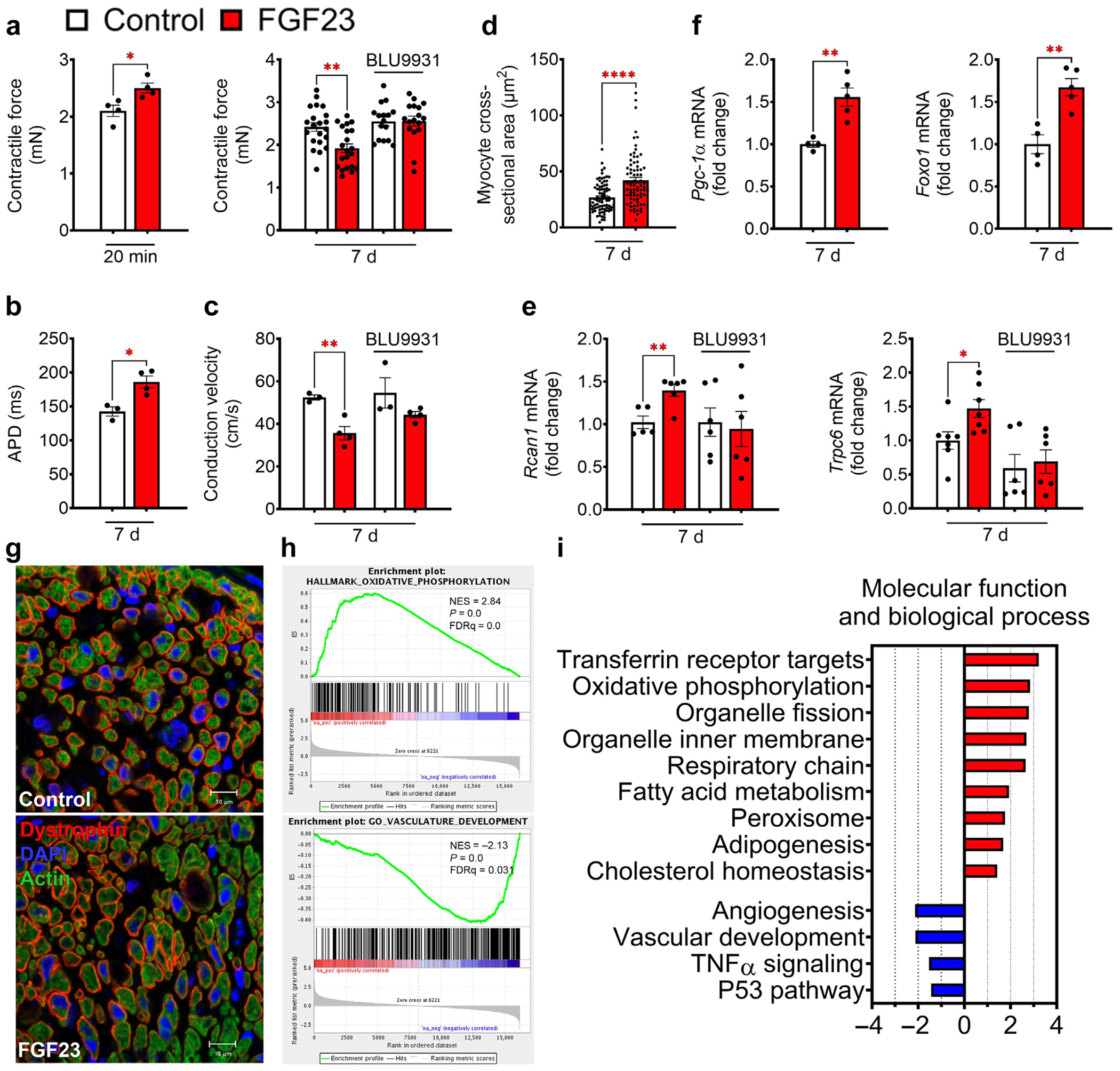
Fibroblast growth factor receptor (FGFR) 4 regulates metabolic transcription and hypertrophy in bioengineered cardiobundles. (**a**) Treatment of neonatal rat ventricular myocyte cardiobundles with fibroblast growth factor (FGF) 23 for 20 minutes significantly increased contractile force, whereas 7 days of chronic treatment led to a significant reduction in contractile force that could be rescued by coapplication of BLU9931, a selective FGFR4 inhibitor. (**b**) Electrophysiological function was evaluated by pacing of cardiobundles and application of Di-4-ANEPPS (6-[2-(N,N-Dibutylamino)naphthyl]ethenyl-4′-pyridinium propanesulfonate) as voltage-sensitive dye. Chronic exposure of cardiobundles to FGF23 lead to significantly longer action potential durations. (**c**) FGF23-treated bundles exhibited significantly lower conduction velocity that was normalized after coapplication of BLU9931. Besides functional changes, chronic FGF23 treatment also led to cardiobundle hypertrophy, indicated by the (**d,g**) significant increase in cross-section and (**e**) increased expression of hypertrophic mRNA markers *Rcan1* and *Trpc6*. Increased expression of *Rcan1* and *Trpc6* was blocked by parallel treatment with BLU9931. (**f**) Metabolic transcription factors that were increased in chronic kidney disease mice also increased in cardiobundles after FGF23 treatment. (**g**) Representative images of cardiobundles indicate cellular hypertrophy after FGF23 treatment by increased myocyte cross-sections. Bars = 10 μm. (**h**) Gene set enrichment analysis of control and FGF23-treated cardiobundles showed an enrichment of metabolic pathways, particularly fatty acid metabolism, adipogenesis, and cholesterol homeostasis. (**i**) Additional enrichment was detected in pathways related to mitochondrial function, such as oxidative phosphorylation, respiratory chain, organelle fission, and organelle inner membrane. Downregulated pathways after FGF23 treatment include angiogenesis, vascular development, tumor necrosis factor (TNF)-α signaling, and P53. Bar graphs represent mean ± SEM with individual values included in the graph. n ≥ 3 for all experiments. *P < 0.05, **P < 0.005, ****P < 0.0001. APD, action potential duration; DAPI, 4′,6-diamidino-2-phenylindole; ES, enrichment score; FDR, false discovery rate; NES, normalized enrichment score. To optimize viewing of this image, please see the online version of this article at www.kidney-international.org.

To investigate whether elevated FGF23-FGFR4 signaling might contribute to the cardiac metabolic remodeling observed in CKD, we studied cardiobundles, which are 3-dimensional multicellular cylindrical tissues bioengineered from NRVMs and fibroblasts. Cardiobundles spontaneously contract and exhibit mature functional properties similar to postnatal rat myocardium.^26^ Acute treatment of cardiobundles with FGF23 (20 minutes) significantly increased contractility compared with vehicle, as reported previously (Figure 4a).^27^ In contrast, chronic FGF23 treatment (7 days) significantly decreased contractility; this effect was blocked by cotreatment with BLU9931, a small-molecule isoform-specific inhibitor of FGFR4 (Figure 4a), confirming the specific effects of FGF23-FGFR4 activation.

To assess electrophysiological function, cardiobundles were paced with a voltage-sensitive dye, followed by optical mapping of the action potential propagation. Chronic FGF23 treatment prolonged action potential duration compared with controls (Figure 4b). Conduction velocity was ~32% slower in FGF23-treated versus vehicle-treated cardiobundles, an effect that was attenuated by BLU9931 (Figure 4c). The selected dose of BLU9931 had no effects on contractile force or conduction velocity of control cardiobundles (Figure 4a and c). To investigate possible direct effects of increased adenine concentrations or increased phosphate levels in CKD on cardiomyocytes, contractile force and frequency of engineered heart tissue were evaluated (Supplementary Figure S3A and B). Incubation of engineered heart tissue with adenine (20 μM) for 6 days had no sustained effects on contractile frequency or force; however, incubation with phosphate (1 mM) showed some reduction in contractile force from day 4 onwards, and frequency was unchanged (Supplementary Figure S3A and B). These results confirm specific FGF23-FGFR4–mediated effects.

In cardiobundles, 7 days of FGF23 treatment stimulated hypertrophic growth indicated by increased cross-sectional area of individual myocytes and elevated mRNA expression of the hypertrophic markers *Trpc6* and *Rcan1* (Figure 4d, e, and g); cotreatment with BLU9931 blocked these effects. Similar to the results from CKD mice, mRNA expression of metabolic transcription factors *Pgc-1α* and *Foxo1* increased in FGF23-treated cardiobundles (Figure 4f).

To investigate the mechanism of FGF23-FGFR4–induced cardiac remodeling, we profiled changes in gene expression of cardiobundles subjected to 7 days of FGF23 treatment using RNA sequencing. Highly enriched metabolic pathways on FGF23 treatment included fatty acid metabolism, adipogenesis, and cholesterol homeostasis (Figure 4h). In addition, FGF23-treated cardiobundles showed strong enrichment in molecular processes related to mitochondrial structure and function, including oxidative phosphorylation, respiratory chain, organelle fission, and organelle inner membrane (Figure 4i). Taken together, these results suggest that FGF23 can induce *in vitro* changes in cardiac tissue that parallel those observed in mice with CKD, and that these effects are directly mediated by FGFR4 activation.

### FGF23-FGFR4 alter mitochondrial function in cultured cardiomyocytes

**Figure 5 |:**
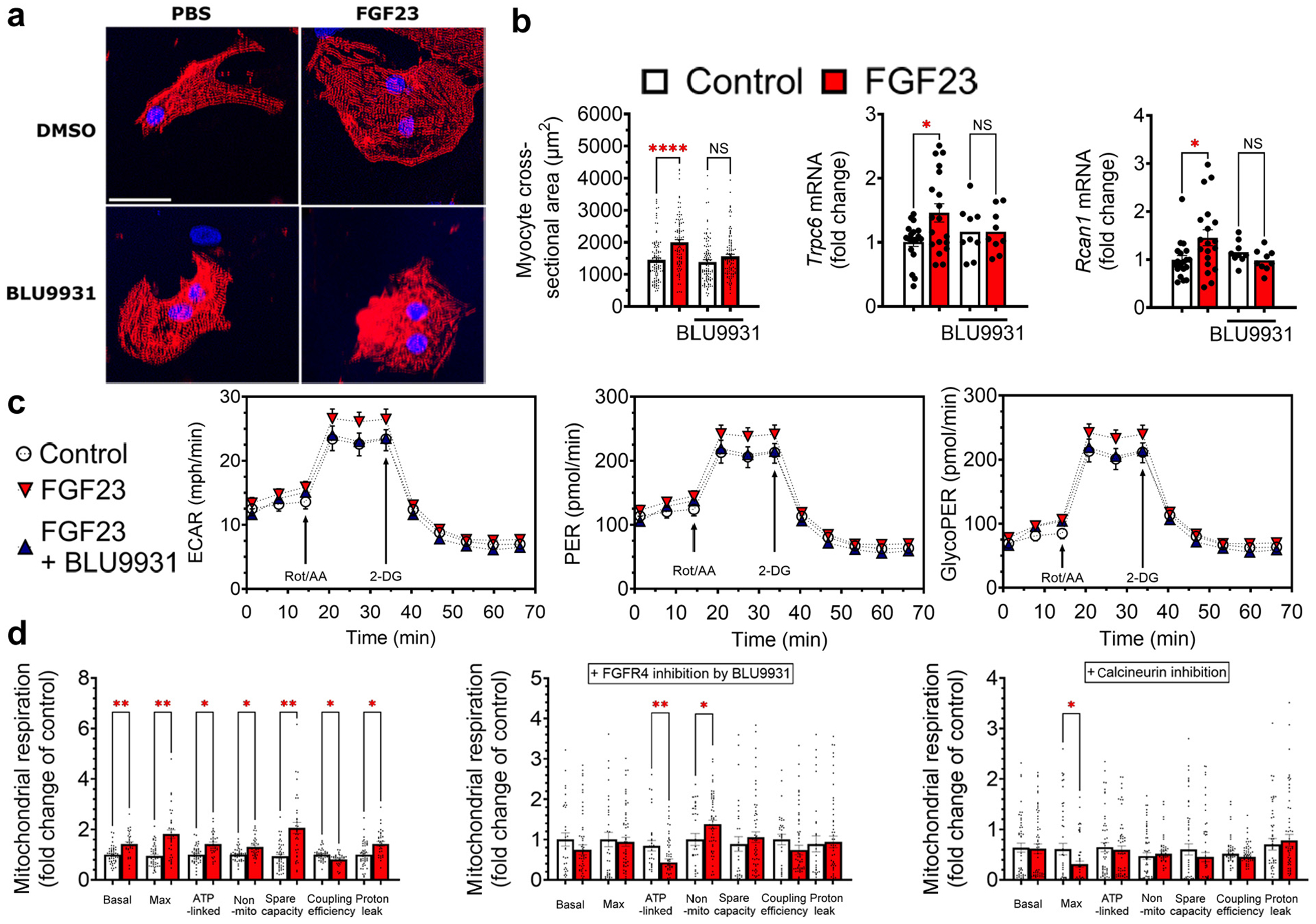
Fibroblast growth factor receptor (FGFR) 4 mediates metabolic remodeling in cultured cardiomyocytes. (**a,b**) Cultured neonatal rat ventricular myocytes (NRVMs) responded to 48 hours of fibroblast growth factor (FGF) 23 treatment with significant hypertrophy, indicated by increased cross-sectional area and expression of prohypertrophic markers. Prohypertrophic mRNA expression and cellular hypertrophy could be mitigated by parallel treatment with the FGFR4-specific inhibitor BLU9931. (**a**) Bar = 30 μm. (**c**) NRVMs treated with FGF23 for 1 hour, before observable hypertrophy takes place, were analyzed in a Seahorse XF analyzer for extracellular acidification rate (ECAR), elevated total proton efflux rates (PERs), and glycolysis-specific PER (GlycoPER). ECAR was significantly higher in FGF23-treated cells, which could be reduced to control levels by BLU9931. PER showed elevated basal and compensatory glycolysis on FGF23 treatment; glycolysis-specific proton efflux was also increased. These FGF23-mediated effects were blocked by BLU9931 application. (**c**) Graphs represent 3 independent experiments. (**d**) Seahorse mitochondrial stress test assay showed increased basal and maximal mitochondrial respiration after FGF23 treatment of NRVMs. Adenosine triphosphate (ATP) production-linked, spare respiratory capacity and nonmitochondrial oxygen consumption rate increased in parallel after FGF23 treatment. The significant decrease in coupling efficiency and the increased proton leak indicate uncoupling of substrate oxidation and ATP synthesis after 1 hour of FGF23 treatment. Application of BLU9931 or the calcineurin inhibitor, cyclosporin A, prevented the changes to mitochondrial function caused by FGF23. Bar graphs represent mean ± SEM and individual values included in the graph. n ≥ 9 for all experiments. *P < 0.05, **P < 0.005, ****P < 0.0001. DMSO, dimethylsulfoxide; Max, maximum; NS, not significant; PBS, phosphate-buffered saline. To optimize viewing of this image, please see the online version of this article at www.kidney-international.org.

Next, we determined if FGF23-FGFR4 directly modulates substrate utilization and mitochondrial respiration in cultured cardiomyocytes. NRVMs were treated with FGF23, with and without BLU9931, for 48 hours. FGF23 induced hypertrophy of NRVMs, as determined by significant increases in the area of immunolabeled cells and elevated mRNA expression of the hypertrophic markers, *Trpc6* and *Rcan1* (Figure 5a and b), as previously reported.^13^ Cotreatment with BLU9931 blocked hypertrophic growth of cardiomyocytes, whereas treatment with BLU9931 itself had no effects (Figure 5a and b).

Activation of glycolysis is observed in cardiac remodeling, including in advanced CKD when LVH is already established.^48–50^ To determine if glycolysis is directly stimulated by the early increase in FGF23 or increases indirectly in response to cellular hypertrophy, we treated NRVMs with FGF23 for 1 hour, before cellular hypertrophy was present (Supplementary Figure S3C). Using the Seahorse XF analyzer, we evaluated the extracellular acidification rate in NRVMs as an indirect measure of glycolysis (Figure 5c). Extracellular acidification rate was significantly higher in FGF23-treated versus control cells; this effect was abolished by BLU9931 (Figure 5c), which had no effect on its own (data not shown). Next, we assessed the glycolytic rate of NRVMs, which removes the contribution of mitochondrial CO_2_ to extracellular acidification rate and allows more accurate measurement of glycolysis. FGF23 significantly increased basal and compensatory glycolysis, as determined by elevated total proton efflux rates and glycolysis-specific proton efflux rates, whereas BLU9931 blocked these effects (glycolysis-specific proton efflux rates; Figure 5c).

To directly examine mitochondrial function in response to FGF23, we used the Seahorse mitochondrial stress test assay. FGF23 significantly increased basal and maximum mitochondrial respiration in NRVMs (Figure 5d). Similarly, adenosine triphosphate production-linked spare respiratory capacity and nonmitochondrial oxygen consumption rate were higher in FGF23-treated cells (Figure 5d). In contrast, FGF23 significantly decreased coupling efficiency, indicating uncoupling of substrate oxidation and adenosine triphosphate synthesis. The observed reduction in coupling efficiency was attributable to a significantly increased proton leak (Figure 5d), which is the predominant mechanism for incomplete coupling.^51^ Pharmacologic inhibition of FGFR4 with BLU9931 and inhibition of calcineurin using cyclosporin A prevented the effects of FGF23 on mitochondrial respiration, implicating the FGFR4–phospholipase Cγ–calcineurin axis in the metabolic effects of FGF23 on glycolysis (Figure 5d).

### Activation of FGFR4 causes cardiac metabolic remodeling independently of CKD

**Figure 6 |:**
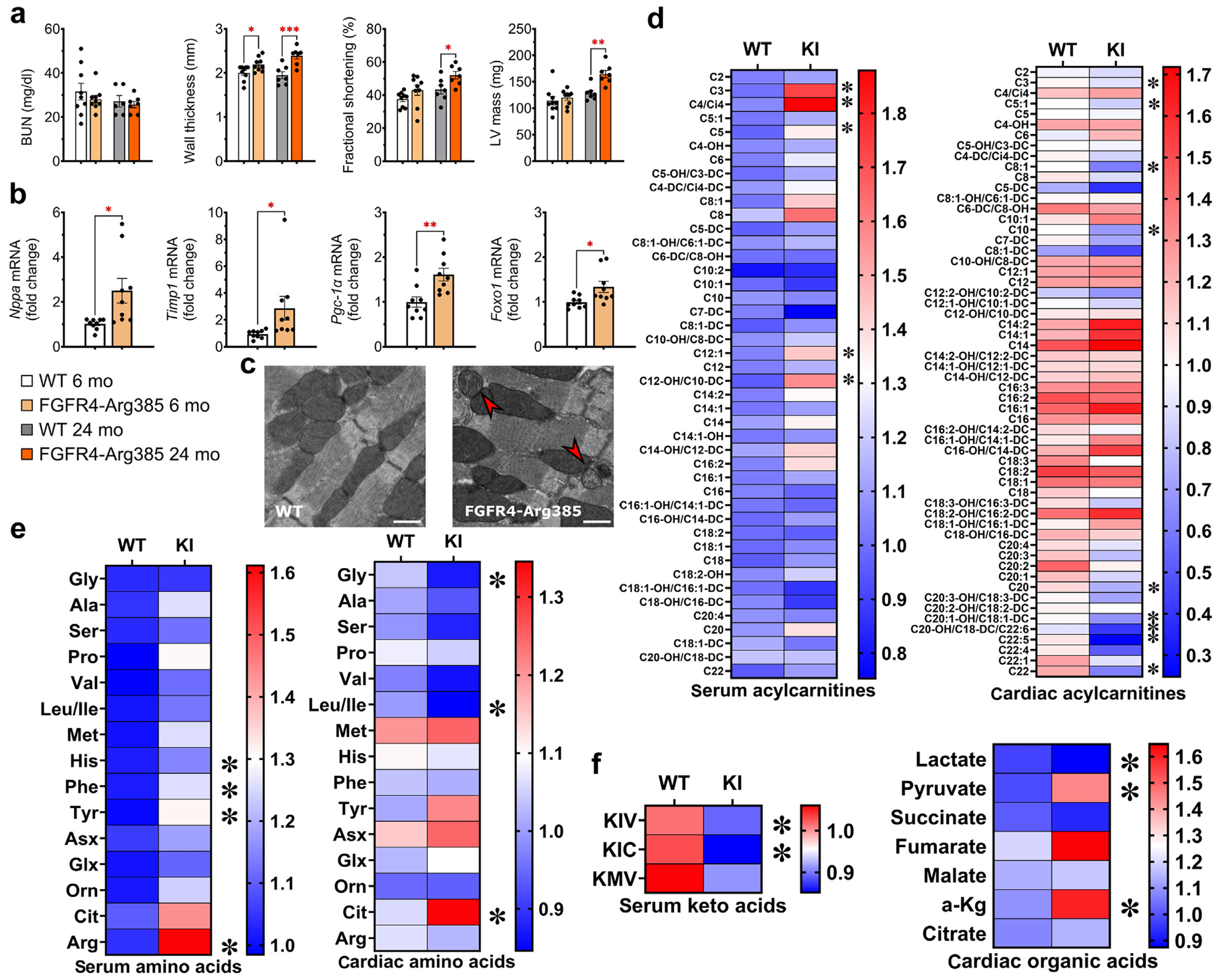
Cardiac function, remodeling, and metabolomics of fibroblast growth factor receptor (FGFR) 4–Arg385 mice in the absence of chronic kidney disease (CKD). FGFR4-Arg385 mice did not have impaired kidney function, as indicated by blood urea nitrogen (BUN) values, but beginning left ventricular hypertrophy (LVH) is detectable by increased wall thickness at 6 months of age. (**a**) By 24 months of age, renal function remained unchanged, and significant LVH/heart failure with preserved ejection fraction was detected in FGFR4-Arg385 mice, indicated by robust structural remodeling and increased fractional shortening. (**b**) mRNA expression levels of remodeling and profibrotic and prohypertrophic markers support initiation of cardiac remodeling at 6 months of age. (**c**) Transmission electron microscopy showed similar changes in the mitochondria of 6-month-old FGFR4-Arg385 mice, as observed in mice with adenine-induced CKD. (**c**) Bar = 500 nm. (**d**) Metabolomic analysis of FGFR4-Arg385 mice (knock-in [KI]) at 6 months of age showed significant increase in some serum acylcarnitines and reduction in cardiac medium- and long-chain acylcarnitines compared with wild-type (WT) animals. (**e**) Similar to WT CKD animals, cardiac citrulline was upregulated, whereas several other amino acids, including leucine and isoleucine, were downregulated. Reduction of serum keto acids was also in line with results obtained from the adenine CKD model. (**f**) Organic acids also showed similar changes with a significant upregulation of pyruvate and a downregulation of lactate. Bar graphs represent mean ± SEM and individual values included in the graph. n ≥ 6 for all experiments. *P < 0.05, **P < 0.005, ***P < 0.0005. KIC, ketoisocaproate; KIV, α-ketoisovalerate; KMV, ketomethylvalerate; LV, left ventricular. To optimize viewing of this image, please see the online version of this article at www.kidney-international.org.

To further test the hypothesis that FGFR4 activation contributes to cardiac metabolic remodeling, we investigated cardiac mitochondria in knock-in mice that express FGFR4-Arg385, which is a gain-of-function mutation of FGFR4.^13,33^ As we reported previously,^13^ there were no differences in kidney function or systemic mineral metabolism between wild-type and FGFR4-Arg385 mice (Figure 6a and Supplementary Figure S4). Six-month-old FGFR4-Arg385 mice developed mild LVH, characterized by increased wall thickness and mRNA expression of hypertrophic and profibrotic markers, but overall LV mass, LV diameters, and systolic and diastolic function were unchanged until 24 months of age, when FGFR4-Arg385 mice manifested overt LVH (Figure 6a and b and Supplementary Figure S4). TEM revealed cardiac mitochondrial morphologic changes in 6-month-old FGFR4-Arg385 mice that were similar to the changes observed in wild-type and Col4a3^–/–^ mice with CKD, including disorganized alignment and swollen mitochondrial cristae (Figure 6c). Analysis of the cardiac and serum metabolome in 6-month-old FGFR4-Arg385 mice demonstrated significant changes in organic acids, several MLACs, branched-chain amino acids, and branched-chain keto acids in a similar pattern as observed in the adenine CKD mice (Figure 6d–f). Taken together, these morphologic and metabolomic data suggest that expression of a constitutively active FGFR4 is sufficient to induce cardiac metabolic remodeling and changes in cardiac mitochondria, which occur before the onset of overt LVH.

### Global deletion of FGFR4 prevents remodeling of the cardiac mitoproteome in CKD

**Figure 7 |:**
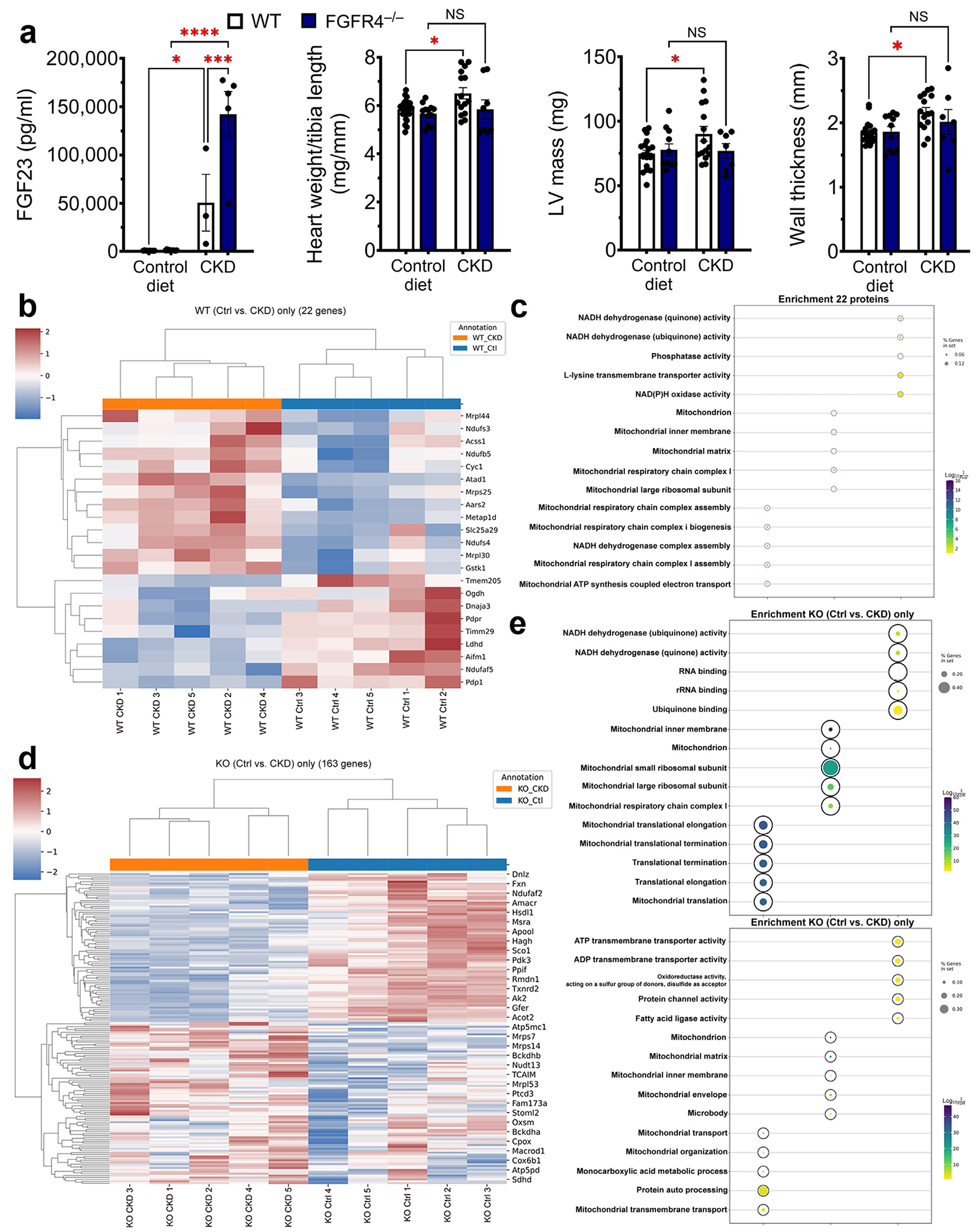
Global deletion of fibroblast growth factor receptor (FGFR) 4 prevents left ventricular hypertrophy (LVH) and changes to cardiac mitoproteome in chronic kidney disease (CKD). (**a**) Mice with global deletion of FGFR4 develop CKD to the same degree as control (Ctrl) mice after 16 weeks of adenine diet, as indicated by the increase in fibroblast growth factor (FGF) 23. (**b**) Wild-type (WT) animals developed LVH at 16 weeks with increased left ventricular (LV) mass, wall thickness, and ratio of heart weight/tibia length. These changes were absent in FGFR4^−/−^ mice. Cardiac mitoproteome ofWT and FGFR4^−/−^ mice was evaluated after 12 weeks of adenine feeding, before overt remodeling is observed. A total of 22 proteins were significantly regulated in WT CKD mice, but were not changed in FGFR4^−/−^ CKD mice, with 9 proteins downregulated and 13 upregulated. (**c**) Analysis showed enrichment in pathways connected to mitochondrial respiration and function. (**d**) Additionally, 163 proteins were identified that were only regulated in FGFR4^−/−^ CKD mice, with 83 downregulated and 57 upregulated proteins. (**e**) Enrichment analysis showed a partial normalization of mitochondrial proteins in FGFR4^−/−^ mice. Bar graphs represent mean ± SEM and individual values included in the graph. n ≥ 3 for all experiments. *P < 0.05, ***P < 0.0005, ****P < 0.0001. ADP, adenosine diphosphate; ATP, adenosine triphosphate; KO, knockout; NADH, reduced nicotinamide adenine dinucleotide; NAD(P)H, reduced nicotinamide adenine dinucleotide phosphate; NS, not significant.

Global deletion of FGFR4 (FGFR4^−/−^) protects mice from LVH caused by chronic high-phosphate diet.^27^ To test if FGFR4 deletion also protects against LVH in CKD, we subjected FGFR4^−/−^ mice to 16 weeks of adenine diet. As reported previously,^36^ all mice developed CKD, with elevations in serum blood urea nitrogen and FGF23 (Figure 7a and Supplementary Figure S5). Control, but not FGFR4^−/−^, mice developed pathologic cardiac remodeling (Figure 7a) and elevated cardiac expression of prohypertrophic (*Nppb*) and profibrotic (*Fn1*) markers (Supplementary Figure S5). Cardiac mRNA expression of metabolic transcription factor *Pgc-1α* was upregulated in control but not FGFR4^−/−^ mice (Supplementary Figure S5).

We isolated mitochondria from the hearts of wild-type mice and FGFR4^−/−^ littermates with CKD and assessed the cardiac mitoproteome with liquid chromatography–mass spectrometry. The 9 proteins that were significantly downregulated and the 13 that were significantly upregulated in wild-type CKD mice were unchanged in FGFR4^−/−^ CKD mice, including proteins of mitochondrial respiration and function (Figure 7b and c). In addition, 83 proteins were downregulated and 57 were upregulated only in FGFR4^−/−^ CKD mice; enrichment analysis of these differentially expressed proteins suggests that deletion of FGFR4 upregulates pathways related to reduced nicotinamide adenine dinucleotide activity, mitochondrial ribosomes, and mitochondrial translation and downregulates pathways linked to adenosine triphosphate transport, protein channel, and fatty acid activity (Figure 7d and e). Taken together, these results indicate that global deletion of FGFR4 attenuates pathologic changes in cardiac mitochondrial composition in CKD.

### FGF23-FGFR4 signaling mediates cardiac metabolic remodeling in adenine-induced CKD

**Figure 8 |:**
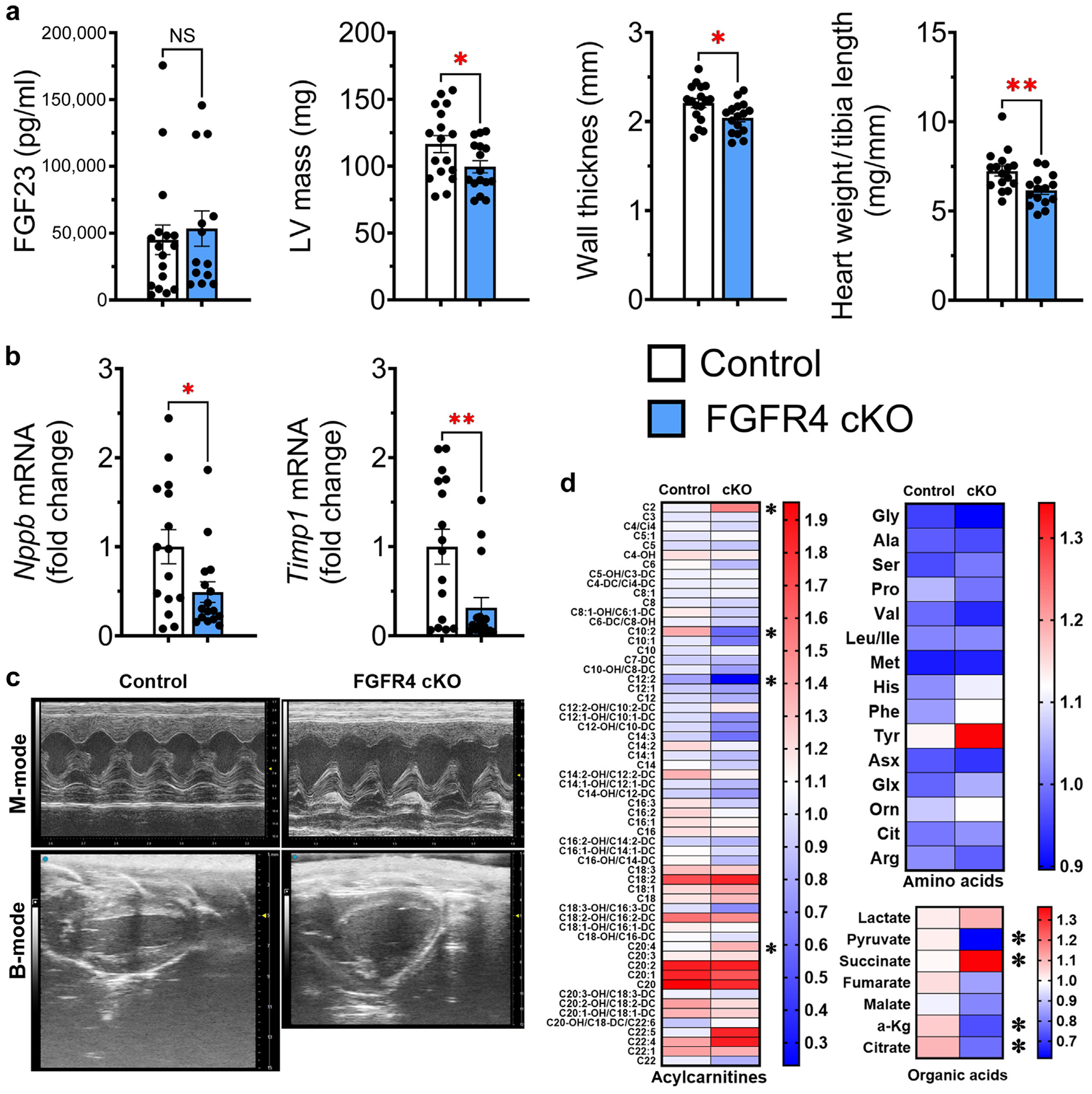
Cardiomyocyte expression of fibroblast growth factor receptor (FGFR) 4 mediates metabolic remodeling in adenine-induced chronic kidney disease. After 16 weeks of adenine diet, control animals and alpha-myosin heavy chain^MerCreMer^–FGFR4^flox^ (FGFR4 conditional knockout [cKO]) mice developed kidney damage to a similar degree with elevated fibroblast growth factor (FGF) 23. (**a**) Left ventricular (LV) mass, wall thickness, and ratio of heart weight/tibia length were significantly lower in FGFR4 cKO mice than control animals. (**b**) Cardiac-specific deletion of FGFR4 also significantly reduced the cardiac expression of prohypertrophic and profibrotic markers. (**c**) Echocardiography showed no structural remodeling in the hearts of FGFR4 cKO mice, whereas hearts of control animals showed significant wall thickening and remodeling. (**d**) Analysis of the cardiac metabolome showed elevation of a greater number of medium- and long-chain acylcarnitines when compared with control animals. Expression levels of organic acids indicate a normalization of glucose use in FGFR4 cKO mice and reduction in cardiac pyruvate and citrate concentrations. Amino acids were unchanged between groups. Bar graphs represent mean ± SEM and individual values included in the graph. n ≥ 15 for all experiments. *P < 0.05, **P < 0.005. To optimize viewing of this image, please see the online version of this article at www.kidney-international.org.

To determine the specific role of cardiac FGFR4 in cardiac metabolic remodeling in CKD, we created mice with inducible cardiomyocyte-specific deletion of FGFR4 (α-MHC^MerCreMer^-FGFR4^flox^). α-MHC^MerCreMer^-FGFR4^flox^ (FGFR4 conditional knockout [cKO]) mice do not develop LVH in response to repeated short-term (5 days) FGF23 injections.^14^ FGFR4 cKO mice were injected with tamoxifen, and cardiac FGFR4 expression was evaluated 10 days later. *Fgfr4* mRNA expression was significantly reduced in the hearts but not the kidneys of FGFR4 cKO mice compared with controls, confirming cardiac-specific deletion of FGFR4 (Supplementary Figure S6). After 16 weeks of adenine diet, all mice developed kidney injury to the same degree (Figure 8a and Supplementary Figure S6). Echocardiography revealed significantly lower LV mass, wall thickness, and heart weight–to–tibia length ratio in FGFR4 cKO mice versus controls (Figure 8a and c). In addition, cardiac mRNA expression of prohypertrophic and profibrotic markers was significantly decreased in FGFR4 cKO mice versus controls (Figure 8b). Control, but not FGFR4 cKO, mice developed diastolic dysfunction (Figure 8c and Supplementary Figure S6).

We analyzed the cardiac metabolome of α-MHC^MerCreMer^-FGFR4^flox^ mice and littermate controls with CKD (Figure 8d). Cardiomyocyte-specific deletion of FGFR4 elevated cardiac levels of MLACs. Although amino acids and branched-chain amino acids remained mostly unchanged, expression levels of organic acids suggested normalization of cardiac pyruvate, succinate, and lactate concentrations. Taken together, these results indicate that blocking cardiac FGFR4 attenuates cardiac metabolic remodeling in CKD.

## DISCUSSION

We demonstrate that cardiac mitochondrial dysfunction and metabolic remodeling are early complications of CKD that occur before structural cardiac remodeling that ultimately progresses to LVH and heart failure. Using a combination of *in vitro* techniques, 2 distinct models of CKD, and gain-of-function and loss-of function genetic mouse models, we further identify FGF23-FGFR4 activation as a potential mechanism of cardiac mitochondrial dysfunction and metabolic remodeling in CKD.

Few prior studies investigated cardiac metabolism in CKD. Those that did mostly focused on single metabolic pathways after structural heart changes were already evident.^49,50,52–54^ The results of this study bridge the cardiac pathogenic gap between the early onset of CKD and the later development of significant structural cardiac changes. We report that early cardiac metabolic changes are upstream of structural cardiac remodeling mechanisms rather than being secondary effects of hypertrophy. Our findings are supported by a previous study that identified cardiac pathology in otherwise asymptomatic patients with end-stage kidney disease by tracing changes in cardiac fatty acid metabolism with single-photon emission computed tomography.^52^ Consistent with previous findings by other groups, we observed changes to fatty acid metabolism with a compensatory increase in glucose use, but we now show that these changes are present even before structural remodeling is detected.^49,50^ Our results also align with recent reports that compared cardiac metabolites from patients with HFpEF, patients with heart failure with reduced ejection fraction, and different animal models.^55,56^

Our *in vitro* experiments using cardiobundles and NRVMs revealed that FGF23 induces contractile, electrical, and metabolic dysfunction that was prevented by a specific small-molecule inhibitor of FGFR4. Pathway enrichment analysis of cardiobundles treated with FGF23 identified processes related to peroxisomal physiology and transferrin receptor biology, highlighting a possible mechanistic link between FGF23 and iron homeostasis, as has been reported previously.^57,58^ These results further underscore the role of FGF23 in driving cardiac dysfunction through distinct molecular pathways.

Cardiac mitochondria regulate cardiac energy homeostasis and metabolism. Our analysis of cardiac mitochondria using TEM, proteomics, and respirometry indicates that CKD causes substantial changes to cardiac mitochondrial structure and function before structural changes of the heart are evident. Similarly, we report that FGF23 induces mitochondrial dysfunction *in vitro*, characterized by increased mitochondrial respiration, aligning with the respirometry findings in CKD mouse models. These observations are consistent with previous studies that describe pathologic mitochondria in a rat model of CKD using high-resolution imaging. Although reduced cardiac oxidative phosphorylation is a hallmark of many heart failure studies,^59,60^ increased mitochondrial respiration has also been reported in right ventricular heart failure following pulmonary hypertension.^61–63^ Given that CKD is associated with premature aging, it is noteworthy that aged mitochondria display a similar phenotype consisting of enhanced oxidative respiration and proton leak.^62^

Functionally, our results indicate that FGFR4-mediated increases in mitochondrial respiration may act as a retrograde signaling mechanism that drives glycolysis and adaptive changes in oxidative catabolism.^64^ The pathologic increases in basal respiration, coupled with sustained proton leak, likely exacerbate bioenergetic stress,^65^ burden the mitochondrial work load, and ultimately lead to widespread mitochondrial injury and reduced respiratory efficiency. Our data on mitochondrial respiration contrast with a recent study using a different model of Alport syndrome, which reported decreased respiration in permeabilized cardiac tissue.^66^ We hypothesize that these discrepancies arise from the more advanced kidney disease and subsequent heart failure analyzed in that report.^66^

Considering a recent publication implicating endogenous adenine in CKD progression and the well-established harmful effects of elevated serum phosphate levels in CKD, we tested whether these factors might directly affect cardiomyocytes independent of FGF23 signaling.^27,44^ In engineered heart tissue, adenine treatment did not impact contractile frequency or force, suggesting no direct cardiac effects. Increased phosphate concentrations led to modest alterations in contractile force but did not affect contraction frequency. These findings align with earlier studies indicating that phosphate can exert direct effects on myocytes, such as modulating metabolic gene expression in myotubes, and eventually promote hypertrophic cardiomyocyte growth.^67,68^ Although intriguing, the molecular mechanisms underlying these phosphate-induced effects on cardiomyocytes remain poorly characterized, and the *in vivo* relevance is uncertain. Mice expressing constitutively active FGFR4 developed cardiac remodeling despite normal serum phosphate levels, whereas global and cardiac-specific FGFR4 knockout mice were protected from cardiac remodeling despite CKD-associated hyperphosphatemia. These results mirror our observations in a previous study on cardiac remodeling in the 5/6 nephrectomy rat model of CKD in which inhibition of the FGF23–FGFR4–calcineurin–nuclear factor of activated T cells signaling pathway prevented cardiac remodeling without altering serum phosphate levels.^13,15,16,27^ Taken together, these findings argue against a major FGF23-independent role of phosphate in driving heart failure in CKD, but further studies are warranted to explore potentially toxic interactions between elevated phosphate and FGF23, as occurs in CKD.

Genetic activation of FGFR4 caused HFpEF in the absence of kidney injury, elevated FGF23, or changes in serum phosphate in our study. Similar to CKD, metabolic changes manifested before overt LVH in mice with constitutively activated FGFR4. Because the cardiac metabolome of wild-type CKD mice and FGFR4-Arg385 knock-in mice was similar, we hypothesized that FGF23-FGFR4 signaling contributes to regulation of cardiac metabolism *in vivo*. This hypothesis is strongly supported by our finding that LVH and HFpEF were attenuated and cardiac metabolism was normalized in mice with CKD overlaying global or cardiomyocyte-specific deletion of FGFR4 compared with mice with CKD and intact cardiac FGFR4.

Limitations of this report include the lack of metabolic flux studies. Our static metabolomic results only provide a snapshot on cardiac metabolic pathways and do not allow a full interpretation of cardiac glycolysis and fatty acid metabolism in CKD. Moreover, we currently do not know how FGFR4 mediates its downstream metabolic effects. Additional experiments will be needed to elucidate the pathway from FGFR4 to mitochondrial dysfunction and whether it includes the phospholipase Cγ–calcineurin signaling cascade, as indicated by the increase in *Trpc6* and *Rcan1* expression in cardiobundles that we observed. Acute and chronic changes in blood pressure were not analyzed for this study, nor did we investigate how vascular or systemic hemodynamic alterations might influence cardiac remodeling in our respective models. However, previous studies have shown that both adenine-fed mice and Col4a3^−/−^ mice develop hypertension.^43,45,69,70^ Importantly, cardiac-specific FGFR4 knockout mice and 5/6 nephrectomized rats treated with an FGFR4 blocking antibody exhibit protection against cardiac remodeling despite developing elevated blood pressure.^27^

Recently, renal glycolysis has been identified as a mammalian phosphate sensor and, thus, energy metabolism serves as a critical regulator of phosphate homeostasis that controls osseous FGF23 secretion via glycerol-3-phosphate.^71,72^ Our results suggest a “metabolic loop” whereby FGF23 itself exerts direct metabolic effects. Our findings that FGF23 mediates cardiac metabolic remodeling and mitochondrial dysfunction support the need to develop inhibitors of FGFR4 or its downstream effectors to prevent adverse cardiac metabolic remodeling and the future development of LVH and HFpEF.

## Supplementary Material

supplementary materials

Supplementary material is available online at www.kidney-international.org.

## Figures and Tables

**Table 1 |:** TaqMan probes used for mRNA quantification

Target	Assay identifier
*Eukaryotic 18S rRNA*	4352930E
*FGFR4*	Mm01341851_g1
*Fib*	Mm01256744_m1
*Foxo1*	Mm00490672_m1
*Myh6*	Mm00440359_m1
*Myh7*	Mm00600555_m1
*Nppa*	Mm01255747_g1
*Pgc-1α*	Mm01208835_m1
*Rcan1*	Rn00596606_m1
*Timp1*	Mm00441818_m1
*Trpc6*	Mm01176083_m1
*β2-Microglobulin*	Mm00437762_m1

## Data Availability

Data analyzed in metabolomic and genomic studies of this study can be accessed here https://figshare.com/s/3ca8b2ec6255dc640df7. Data of proteomics analysis are available at https://massive.ucsd.edu/ProteoSAFe/static/massive.jsp under the identifier MSV000094087. Username “MSV000094087_reviewer” and the reviewer password “CKD” will grant access to the data until they are made publicly available on acceptance of the manuscript. All other data presented in this article will be made available to other researchers on request to the corresponding author.

## Translational Statement

Cardiovascular disease is the leading cause of death in patients with chronic kidney disease (CKD). Elevated fibroblast growth factor (FGF) 23 levels contribute to structural cardiac remodeling and heart failure. Our experimental studies elucidate the metabolic signature of the uremic heart, highlight the role of cardiac metabolism and mitochondrial dysfunction in heart failure due to CKD, and mechanistically indicate that the FGF23–FGF receptor (FGFR) 4 axis drives cardiac metabolic remodeling in CKD. Our results suggest that pharmacologic blockade of FGFR4 might prevent early metabolic alterations in the heart that culminate in heart failure in patients with CKD.
