# Supplementary material for: Fibroblast growth factor 23 and fibroblast growth factor receptor 4 promote cardiac metabolic remodeling in chronic kidney disease
Source: Kidney Int. Author manuscript; Available in PMC 2025 Dec 31. (PMC12755233; doi:10.1016/j.kint.2025.01.024)
Supplement: supplementary materials [file NIHMS2126822-supplement-supplementary_materials.pdf]

Supplement to:

Fibroblast Growth Factor (FGF) 23 and FGF Receptor 4 promote cardiac metabolic remodeling in chronic kidney disease

Michaela A.A. Fuchs[1] PhD, Emily J. Burke[1] B.Sc, Nejla Latic[1,2] PhD, Susan L. Murray[1] MD, Hanjun Li[3] PhD, Matthew A. Sparks[1] MD, Dennis Abraham[4] MD, Hengtao Zhang[4] PhD, Paul Rosenberg[4] MD, Umber Saleem[5,6] PhD, Arne Hansen[5,6] MD, Sara E. Miller[7] PhD, Davis Ferreira[7] PhD, Sonja Hänzelmann[8,9] PhD, Fabian Hausmann[8,9] PhD, Tobias Huber[10,11] MD, Reinhold G. Erben[12] MD, Kelsey Fisher-Wellman[13,14,15] PhD, Nenad Bursac[3,16] PhD, Myles Wolf[1,17,18] MD and Alexander Grabner[1,10,11,17] MD*

Contents:

- Supplemental Methods
- Supplemental Figures: 6
- Supplemental References

Supplemental Methods

Isolation and cultivation of neonatal rat ventricular myocytes

Briefly, the pups were euthanized via rapid decapitation and the hearts were quickly excised and placed in ice-cold calcium- and magnesium-free Hank's Balanced Salt Solution (HBSS). The hearts were then minced and digested with 50 µg/ml trypsin at 4°C for 18-20h. Soybean trypsin inhibitor in HBSS was added, and the tissue was further digested with collagenase (in Leibovitz L-15 medium) under slow rotation (15 rpm) at 37°C for 45min. Cells were released by triturating the suspension 20 times with a standard 10 ml plastic serological pipette and filtering it twice through a cell strainer (70 µm, BD Falcon). Cells were incubated at room temperature for 20min and spun at 75 g for 5min. The cell pellet was resuspended in plating medium Dulbecco's Modified Eagle Medium (DMEM) with 17% Media 199 (Invitrogen), 15% fetal bovine serum (FBS; Invitrogen) and 1% Antibiotic-Antimycotic (Cat.No. 15240096, Gibco). The cell suspension was then plated onto culture plates and incubated at 37°C and 5% CO_2 for at least 2h. Non-myocytes were then removed by differential plating and myocytes were collected and counted using an automated cell counter (Bio-rad). For cell tracings, $3x10^5$ cells per well were grown in 24-well plates on glass slips that were coated with gelatin for 1h at 37°C dissolved in sterile ddH_2O. For seahorse flux analysis, $2x10^4$ cells were grown per well in 96-well plates that were coated with laminin at 10 mg/ml for 1h at 37°C dissolved in PBS. Myocytes were left undisturbed in plating medium and then cultured in maintenance medium (DMEM with 20% Media 199, 1% insulin-transferrin-sodium selenite solution [ITS; Sigma-Aldrich] and 1% Anti/Anti) in the presence of 100 µM 5-bromo-2'-deoxyuridine (BrdU; Sigma-Aldrich). After 4 days, isolated cardiac myocytes were cultured in BrdU-containing maintenance medium in the presence of recombinant murine FGF23 (at 25 and 100 ng/ml) and Heparin (0.2 USP/ml) for different timepoints.

Fabrication of bioengineered cardio-bundles

In brief, NRVMs were isolated and 3.75 x 10^5 cells were embedded in a fibrin-based hydrogel cast into 7 mm x 2 mm troughs of pre-fabricated polydimethylsiloxane (PDMS) molds. Obtained cardio-bundles were then anchored at each end by a porous nylon frame (Cerex® Advanced Fabrics) and removed from troughs to allow free movement in culture media. Cardio-bundles were cultured on a static platform for a total period of 14 days. Full media changes were completed every other day.

Differentiation of human induced pluripotent stem cells to engineered heart tissue and analysis of contraction force and frequency

The expansion of hiPSCs was carried out in Geltrex-coated cell culture vessels using FTDA medium with passaging performed using EDTA. Embryoid bodies were formed in spinner flasks, and cardiac differentiation was induced in Pluronic F 127-coated T175 flasks by sequential addition of growth factors and small molecules. Cardiomyocytes were dissociated by collagenase solution and cardiac differentiation efficiency was assessed by flow cytometry for cardiac troponin T expression. For the generation of engineered heart tissue (EHT), dissociated cardiomyocytes were used to prepare a fibrin-based cell suspension. Agarose slots were created in a 24-well culture plate (Nunc) using 2% agarose and Teflon spacers (EHT Technologies). The cell suspension (97 µl) was mixed with thrombin (3 µl) and transferred into the agarose slot, which contained PDMS posts (EHT Technologies). The EHT plate was incubated at 37°C, 90% humidity, 40% O_2, and 7% CO_2 for 90min. After incubation, PDMS racks with attached EHTs were carefully removed from the agarose slots by adding 200-300 ml of EHT medium and placed into a new culture plate with 1.5 ml of EHT medium per well. The EHTs were cultured in EHT medium for 30 days, with medium changes on Mondays, Wednesdays, and Fridays.[1–3] To quantify contraction force and frequency, video was recorded for each EHT at a frame rate of 100 frames

per second. Sodium phosphate buffer was prepared by mixing NaH2PO4 (Cat.No. S9638, Merck), and Na2HPO4 (Cat.No. 567550, Merck), and pH was adjusted to 7.2 using NaOH. To assess the effects of adenine (Cat.No. 1840, Merck) and phosphate, EHTs were recorded after a 2h incubation in freshly prepared EHT medium, with or without the compounds**.** Recordings were performed in EHT medium with 10% horse serum and values presented are normalized to time- and vehicle-control conditions. A custom algorithm was used to automatically detect the top and bottom boundaries of the EHT and track these positions throughout the contraction cycle. Force was calculated based on the degree of EHT shortening, as well as the elasticity and geometry of the PDMS posts to which the tissues were anchored. The raw data were plotted with force on the y-axis and time on the x-axis, followed by Gaussian filtering and baseline correction.

Immunofluorescence and morphometry of cultured myocytes and cardio-bundles

Briefly, NRVMs were fixed in 2% paraformaldehyde (PFA) (in 5 mg/ml sucrose) for 5min and permeabilized in 1% Triton X-100 (in PBS) for 10min. Coverslips were washed with PBS and blocked for 1h using 1x animal free blocking solution (VectorLabs). The primary sarcomeric α-actinin antibody was used at 1:1000 followed by a secondary Cy3 conjugated antibody at 1:300. To visualize nuclei, fixed cells were incubated with Hoechst for 10mins. Immunofluorescence images were taken on a Leica SP5 inverted confocal microscope with a 40x air objective. As done before, hypertrophic growth of cardio-bundles was analyzed after 7 days of treatment.[4]

Live-cell metabolic analysis

Briefly, NRVM were seeded in Seahorse assay plates at a density of $3x10^4$ / well. NRVM were stimulated with FGF23 (100 ng/ml), Heparin (0.2 USP/ml) and BLU9931 (10 ng/ml) for 60min. One hour before beginning the measurements, the medium was replaced with XF assay medium,

and the cells were incubated for 1h at 37°C without CO_2. The standard protocols for mitochondrial stress and glycolytic rate were performed as previously reported by others.[5]

Mice

Male and female mice were used for this study according to the following sex distribution:

**Study**	**male**	**female**
16 weeks WT CKD		XXX
8 weeks WT CKD	XXX	
12 weeks WT CKD	XXX	XXX
Col4a3$^{-/-}$	XXX	XXX
FGFR4-Arg385	XXX	
FGFR4$^{-/-}$		XXX
α-MHC^{MerCreMer}-FGFR4^{flox}	XXX	XXX

Non-invasive assessment of kidney function

Briefly, the back of each mouse was shaved followed by chemical depilation. Under brief inhalation anesthesia, the NIC kidney sensor was applied directly to the skin of the mouse with adhesive tape followed by i.v. injection of FITC-sinistrin. Conscious mice were then single housed for one hour. The decrease in fluorescent intensity from renal clearance of FITC-sinistrin over one hour allows for the calculation of real glomerular filtration rate.[6–8]

Non-invasive and invasive assessment of cardiac function

Mice were briefly anesthetized with ketamine (100 mg/kg body weight, i.p.). For analysis, both B- and M-mode images were obtained in the short- and long-axis views. Correct positioning of the transducer was ensured using B-mode imaging in the long-axis view before switching to the short-axis view. Image analysis was performed using Vevo LAB software (FUJIFILM VisualSonics).

Biventricular pressure volume loop analyses were performed by Duke's Cardiovascular Physiology Core as described previously.[9]

Mitochondrial respiration

Briefly, minced heart tissue was suspended in ice-cold Buffer A (50 mM MOPS, 100 mM KCl, 1 mM EGTA, 5 mM $MgSO_4$, 2 g/l bovine serum albumin; pH=7.1) and homogenized via a Teflon pestle and borosilicate glass vessel, then centrifuged at 800 x g for 10min at 4°C. Supernatant was centrifuged at 10,000 x g for 10min at 4°C. The mitochondrial pellet was then washed in Buffer B (Buffer A with no bovine serum albumin), transferred to a microcentrifuge tube, and centrifuged again at 10,000 x g for 10min at 4°C. Final mitochondrial pellets were resuspended in 100-150 ml of Buffer B and protein content was determined via the Pierce BCA protein assay.

Respiration media was Buffer C (105 mM K-MES, 30 mM KCl, 10 mM KH_2PO_4, 5 mM $MgCl_2$, 1 mM EGTA, 2.5 g/l bovine serum albumin; pH=7.2), with additions as noted. All respiration experiments were conducted in a 1 ml reaction volume at 37°C. To perform the assay, mitochondria (20 µg) were added to the chamber, followed by ADP (500 µM). Complex I-linked respiration was stimulated with NADH (2 mM), then inhibited with rotenone (Rot; 0.5 µM). Complex II-linked respiration was then stimulated with succinate (S; 10 mM) and inhibited by antimycin A (0.5 µM) to inhibit complex III.

RNA sequencing

RNA sequencing was performed in collaboration with Duke's Center for Genomic and Computational Biology Core Facility. In brief, RNA-seq data was processed using the TrimGalore toolkit[10] which employs Cutadapt[11] to trim low-quality bases and Illumina sequencing adapters from the 3' end of the reads. Only reads that were 20 nt or longer after trimming were kept for further analysis. Reads were mapped to the GRCm38v73 version of the mouse genome and transcriptome[12] using the STAR RNA-seq alignment tool[13]. Reads were kept for subsequent analysis if they mapped to a single genomic location. Gene counts were compiled using the HTSeq tool.[14] Only genes that had at least 10 reads in any given library were used in subsequent analysis. Normalization and differential expression were carried out using the DESeq2[15] Bioconductor[16] package with the R statistical programming environment[17]. The false discovery rate was calculated to control for multiple hypothesis testing. Gene set enrichment analysis[18] was performed to identify gene ontology terms and pathways associated with altered gene expression for each of the comparisons performed.

Proteomics

Frozen mitochondrial pellets were resuspended in 50-100 µl of 5% w/v SDS in 50 mM triethylammonium bicarbonate (TEAB, pH 8.5) followed by probe sonication. Samples were heated for 5min at 80°C followed by centrifugation. Protein concentrations were determined by BCA assay. 50 µg of protein was adjusted to 50 ul with lysis buffer and reduced by heating with 10 mM DTT at 80°C for 10min. Next, reduced thiols were alkylated with 25 mM iodoacetamide at room temperature for 30min. Finally, 18 µg of each sample was processed using an S-Trap micro device (Protifi). Digestion was performed using 1 µg of Sequencing Grade Modified Trypsin (Promega) per sample at 47°C for 1h. After elution from the S-Trap, peptides were lyophilized and resuspended in 30 µl of 1% TFA/ 2% MeCN. A QC pool was made by combining 4.5 µl of each sample.

Quantitative Mass Spectrometry. Quantitative one-dimensional liquid chromatography, tandem mass spectrometry (1D-LC-MS/MS) was performed on 1.25 µl of the peptide digests per sample in singlicate based on an initial loading study. After two conditioning runs with the QC pool, samples were analyzed in a semi- randomized manner, with 4 additional interspersed QC pools. The LC-MS/MS used a nanoACQUITY UPLC system (Waters) coupled to a Q-Exactive HF-X high resolution accurate mass tandem mass spectrometer (ThermoFisher) via a nanoelectrospray ionization source. Briefly the sample was first trapped on a Symmetry C18 180 µm × 20 mm trapping column (5 µl/min at 99.9/0.1 v/v H2O/MeCN) followed by an analytical separation using a 1.7 µm Acquity HSS T3 C18 75 µm × 250 mm column (Waters) with a 90min gradient of 5 to 30% MeCN with 0.1% formic acid at a flow rate of 400 nl/min and column temperature of 55°C. Data collection on the HF-X MS was performed in data-dependent acquisition (DDA) mode with a 120,000 resolution (at m/z 200) full MS scan from m/z 375 to 1600 followed by a target AGC value of 3e6 ions and 50 ms maximum injection time (IT). MS/MS used a Top30 method at 15,000 resolution and with an AGC targeted of 5e4 and max IT of 45 ms, and isolation window of 1.2 m/z and normalized collision energy of 27. Peptides were selected for MS/MS using a minimum AGC target of 2.25e3, charge state filtering (2-5), an apex trigger of 5 to 40s, and a dynamic exclusion of 20s. The total analysis cycle time for each sample injection was approximately 2h.

Following the MS analysis, data was processed using Proteome Discoverer 2.3 (ThermoFisher). Data processing used Minora Feature Detector with min. trace length of 3, max. RT of isotope patterns of 0.2min, and PSM confidence of at least medium. Database searching was performed using Mascot 2.4 using a Swissprot database with mus musculus taxonomy (downloaded on 042219; 17,417 unique sequences) with trypsin specificity, up to 2 missed cleavages, precursor mass tolerance of 5 ppm, fragment mass tolerance of 0.02 Da, static carbamodimethyl(C), variable oxidation(M) and deamidation(NQ). Default percolator settings were used for FDR determination. Consensus steps used the Feature Mapper with RT alignment and a max RT shift

of 5min and min S/N threshold of 1. The precursor ions quantified used unique+razor peptides and intensity precursor abundance. Normalization was to total peptide amount after exclusion of major non-mitochondrial proteins (myosin-6, myosin-7, titin, actin, myosin light chain 3, tropomyosin alpha-3, myosin regulatory light chain 2 and myosin-binding protein c). Protein abundances were calculated using summed peptide abundances, and imputation used replacement of missing values with random values sampled from the lower five percent of detected values. Data was exported for master proteins that met a high confidence (1% peptide and protein) FDR. An unpaired t-test was performed on log2-normalized data in Excel.

Metabolomics

Briefly, amino acids and acylcarnitines were analyzed by flow injection electrospray ionization tandem mass spectrometry and quantified by isotope or pseudo-isotope dilution using methods described previously.[19,20] Samples were spiked with a cocktail of heavy-isotope internal standards (Cambridge Isotope Laboratories; CDN Isotopes) and deproteinated with methanol. Methanol supernatants were dried and esterified with either acidified methanol or butanol for acylcarnitine or amino acid analysis, respectively. Mass spectra for acylcarnitine and amino acid esters were obtained using precursor ion and neutral loss scanning methods, respectively. The spectra were acquired in a multi-channel analyzer (MCA) mode to improve signal-to-noise. The data were generated using a Waters Xevo TQD mass spectrometer equipped with AcquityTM UPLC system and a data system controlled by MassLynx 4.1 operating system (Waters). Ion ratios of analyte to respective internal standard computed from centroided spectra were converted to concentrations using calibrators constructed from authentic aliphatic acylcarnitines and amino acids (Sigma; Larodan) and dialyzed fetal bovine serum (Sigma).

Organic acids were analyzed by capillary gas chromatography/mass spectrometry (GCMS) using isotope dilution techniques employing Trace Ultra GC coupled to ISQ MS operating under

Xcalibur 2.2 (Thermo Fisher Scientific).[21] Briefly, samples were spiked with a mixture of heavy isotope labeled internal standards and the keto acids were stabilized by ethoximation. The samples were acidified and organic acids were extracted into ethyl acetate. The extracts were dried and derivatized with N,O-bis(trimethylsilyl) trifluoroacetamide. The organic acids were quantified using ion ratios determined from single ion recordings of fragment ions which were specific for a given analyte and its internal standard. These ratios were converted to concentrations using calibrators constructed from authentic organic acids (Sigma). Data were analyzed at an adjusted *P* value of 0.1.

Transmission electron microscopy

In brief, hearts were perfused with 2% glutaraldehyde and 4% paraformaldehyde in 0.1 M phosphate buffer (pH 7.4) at a rate of 5 ml/min for 10min to fix the tissue. After perfusion fixation, the hearts were carefully dissected into 1-2 mm^3 small pieces to ensure optimal fixation. The dissected tissue was then immersed in fresh 2.5% glutaraldehyde in 0.1 M Sodium Cacodylate for 1h at room temperature. The tissue was washed three times for 15min with 0.1 M Sodium Cacodylate buffer (pH 7.4) and then post-fixed in 1% osmium tetroxide in 0.1 M Sodium Cacodylate phosphate buffer (pH 7.4) for 2h at room temperature. The tissue was further rinsed three times for 10min with 0.1 M Sodium Cacodylate buffer and then dehydrated in a graded series of acetone and embedded in epoxy resin. Ultrathin sections (70-80 nm) were cut on a LEICA M80 ultramicrotome (Leica) and collected on copper grids. The sections were post-stained with uranyl acetate and lead citrate and then examined with a JEOL 2100 Transmission Electron Microscope (TEM) at an accelerating voltage of 120 kV.

Semiquantitative analysis of mitochondrial morphology

Transmission electron microscopy images with randomized labels were loaded into the ImageJ analysis software with the appropriate pixel to nm scale.[22] Mitochondrial size and number were

measured on each view field by outlining all completely visible mitochondria. Events of obvious mitochondrial damage (i.e. rupture or lysis) were counted on each view field. Average of individual mitochondrial size was calculated for each animal using all counted images of that animal. The number of damaged mitochondria was normalized to the number for view fields counted for each animal. For each animal, at least five view fields were analyzed. Investigators were unblinded after completion of the presented analysis.

Statistical Analysis

Identification of possible statistical outliers was performed by ROUT method (Q=1%) in GraphPad Prism10.

The normalized metabolite abundance tables were log1p-transformed. For differential abundance analysis, each metabolite was tested between control and CKD samples using an univariate linear model, using the Benjamini-Hochberg for multiple testing correction. Metaboanalyst was used to extract enriched metabolic pathways from the differential abundance results.[23]

Enrichment analysis was performed and visualized using gseapy (v1.0.6)[24] using the following databases GO_Cellular_Component_2018', 'GO_Molecular_Function_2018' and 'GO_Biological_Process_2018.

Proteomics heatmaps were visualized using seaborn (v0.11.2).[25]

Supplemental Figure S1- Cardiac functional and mitochondrial parameters of wild-type mice with adenine CKD.

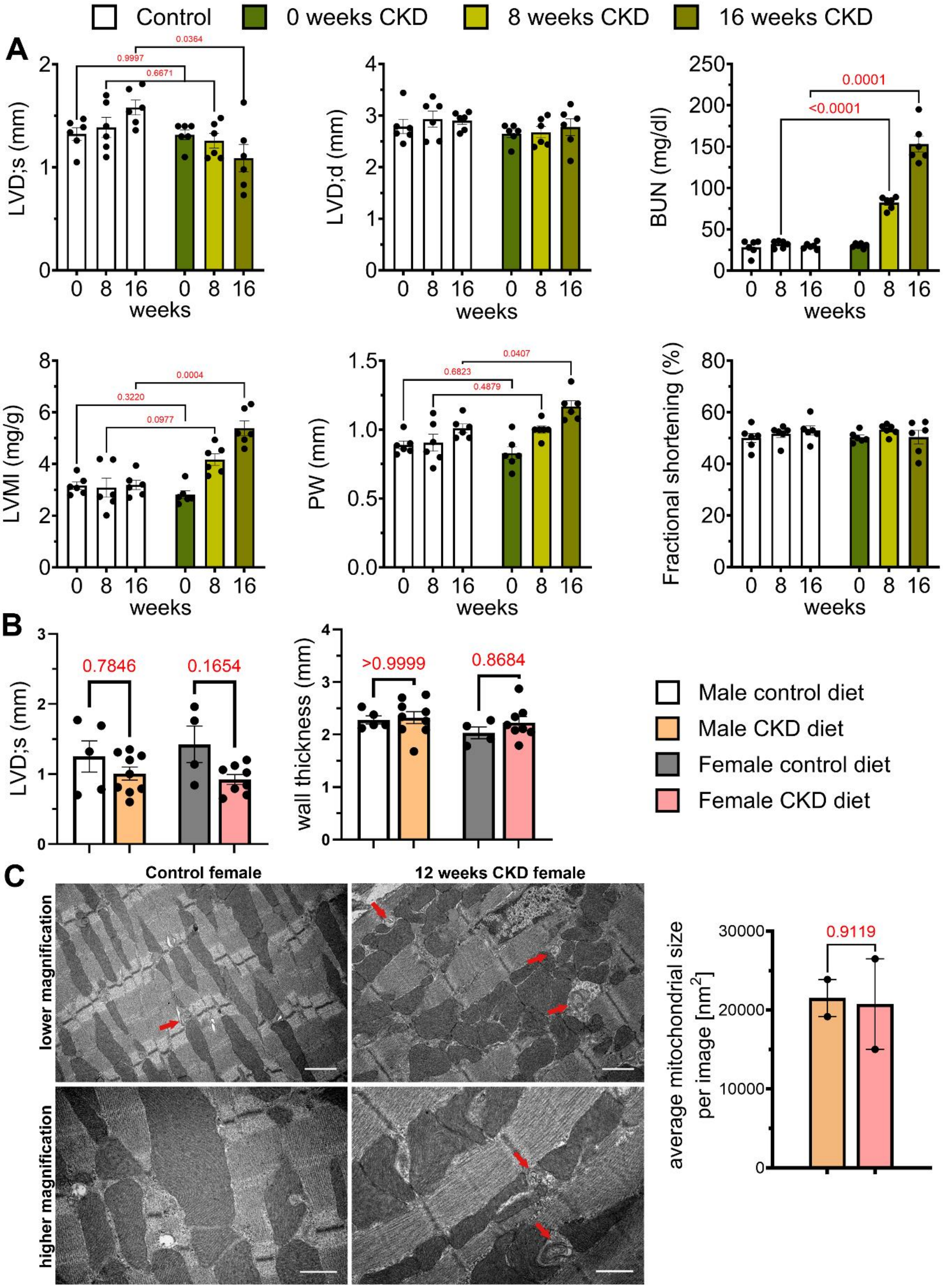

In a preliminary experiment, functional cardiac parameters were measured at different timepoints to investigate the degree of cardiac remodeling with progressive CKD (A). Left ventricular systolic and diastolic diameter (LVD;s and LVD;d) and fractional shortening were not changed until 16 weeks of adenine diet, while BUN was significantly increased by 8 weeks, indicating significant kidney damage. At 16 weeks, LVD;s, LVMI and posterior wall thickness (PW) showed significant cardiac structural remodeling, with preserved function as indicated by unchanged LVD;d and fractional shortening. Detailed sex-specific analysis of cardiac parameters in male and female mice after 12 weeks on the adenine diet showed similar, non-significant, trends indicating similar cardiac pathology, independent of sex in our model at this timepoint (B). Analysis of mitochondria in hearts of male and female mice showed no sex-dependent difference in the morphological changes precipitated by CKD (C). Electron microscopy in the hearts of female mice with 12 weeks adenine induced CKD showed a comparable degree of mitochondrial swelling, misalignment and an increased size of mitochondria as shown in Figure 1.

Bar graphs represent mean ± SEM and individual values included in the graph, *P* values are stated on the bars connecting the respective graphs. Scale bar in C is 1 µm for lower magnification images and 600 nm for higher magnification images.

Supplemental Figure S2 – Cardiac and renal characteristics of Col4a3$^{-/-}$ mice at 20 weeks of age.

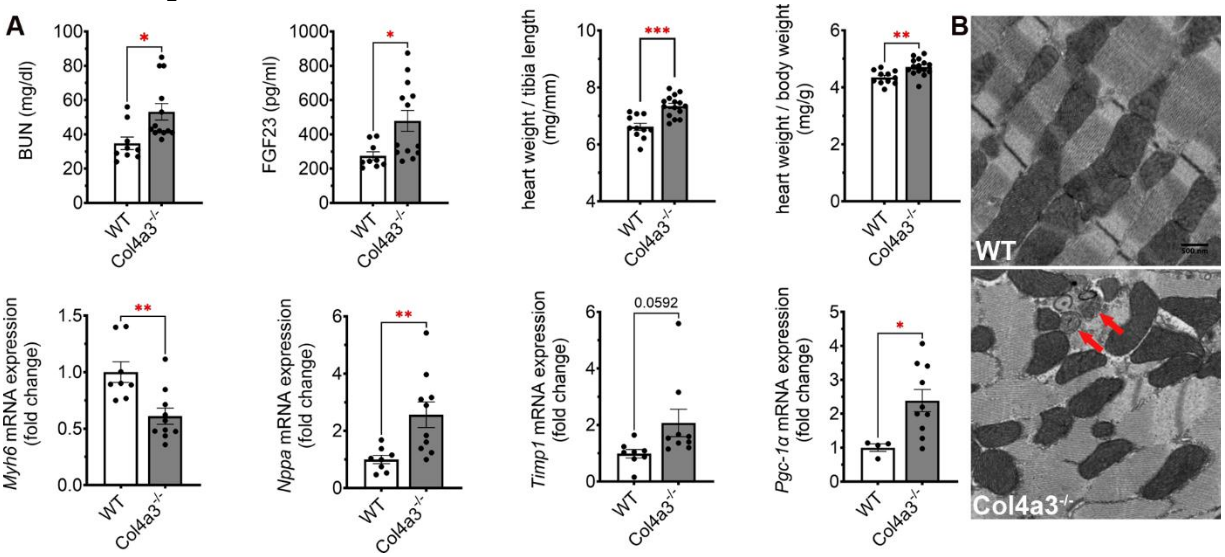

Changes in kidney and cardiac function of C57BLJ/6-Col4a3$^{-/-}$ mice, a genetic model for CKD, and changes in relevant mRNA parameters. Col4a3$^{-/-}$ mice and their respective controls were evaluated at 20 weeks of age, when CKD and beginning LVH manifested as indicated by significant increase of BUN, FGF23, heart weight/tibia length and heart weight/bodyweight. Measurement of pro-hypertrophic, pro-fibrotic and metabolic transcription factors are further evidence of cardiac remodeling (A). Electron microscopy revealed clear changes in mitochondrial morphology similar to that observed in mice with CKD after feeding of an adenine containing diet (B). Changes observed in Col4a3$^{-/-}$ mice were in line with those observed in the adenine model, indicating a generalized mechanism directing the development of LVH, not model specific particularities.

Bar graphs represent mean ± SEM and individual values included in the graph. n ≥ 4 for all experiments. * indicate $P < 0.05$. Scale bar in B is 500 nm.

Supplemental Figure S3 – Effect of adenine and phosphate on engineered heart tissue and markers of cellular hypertrophy in NRVM.

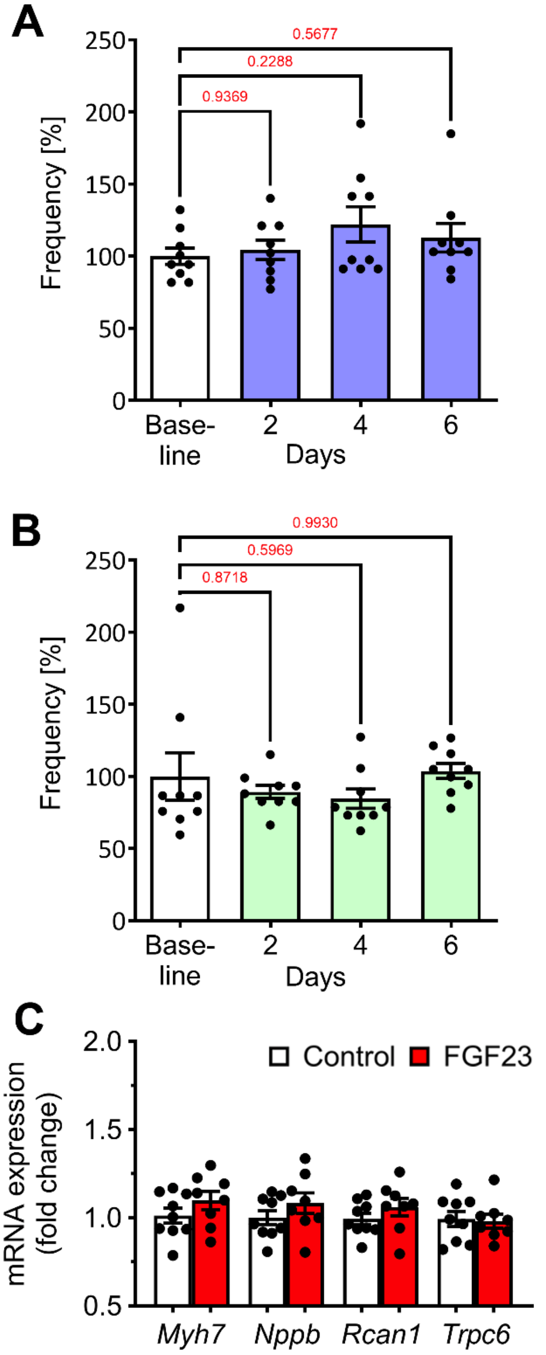

Engineered heart tissues (EHT) derived from human induced pluripotent stem cells were treated with either vehicle, 20 µM adenine (A) or 1 mM phosphate (B) for 6 days to evaluate the direct effect on cardiomyocytes. Treatment with adenine had no effect on the contraction frequency throughout the experimental period. A decrease in contraction force on day 4 in adenine treated

EHT was transient on no longer detectable in EHT treated for 6 days. Prolonged treatment of EHT with phosphate had no effect on contraction frequency, but showed a reduction in the contraction force from 4 days of treatment until day 6.

To determine if glycolysis is directly stimulated by FGF23 or indirectly as a response to cellular hypertrophy, we treated NRVM with FGF23 for 1h, when no signs of cellular hypertrophy were present. At 1h after FGF23 treatment, markers of cellular hypertrophy were not significantly induced compared to vehicle treated cells (C).

Bar graphs represent mean ± SEM and individual values included in the graph. n ≥ 8 for all experiments. Contraction frequency and force measurements were normalized to time- and vehicle-control conditions. Statistical analysis was performed using a repeated-measures one-way ANOVA with Dunnett's multiple comparisons post-test versus baseline where appropriate, or by student’s T-test.

Supplemental Figure S4 – Additional parameters of renal and cardiac function in FGFR4-Arg385 mice.

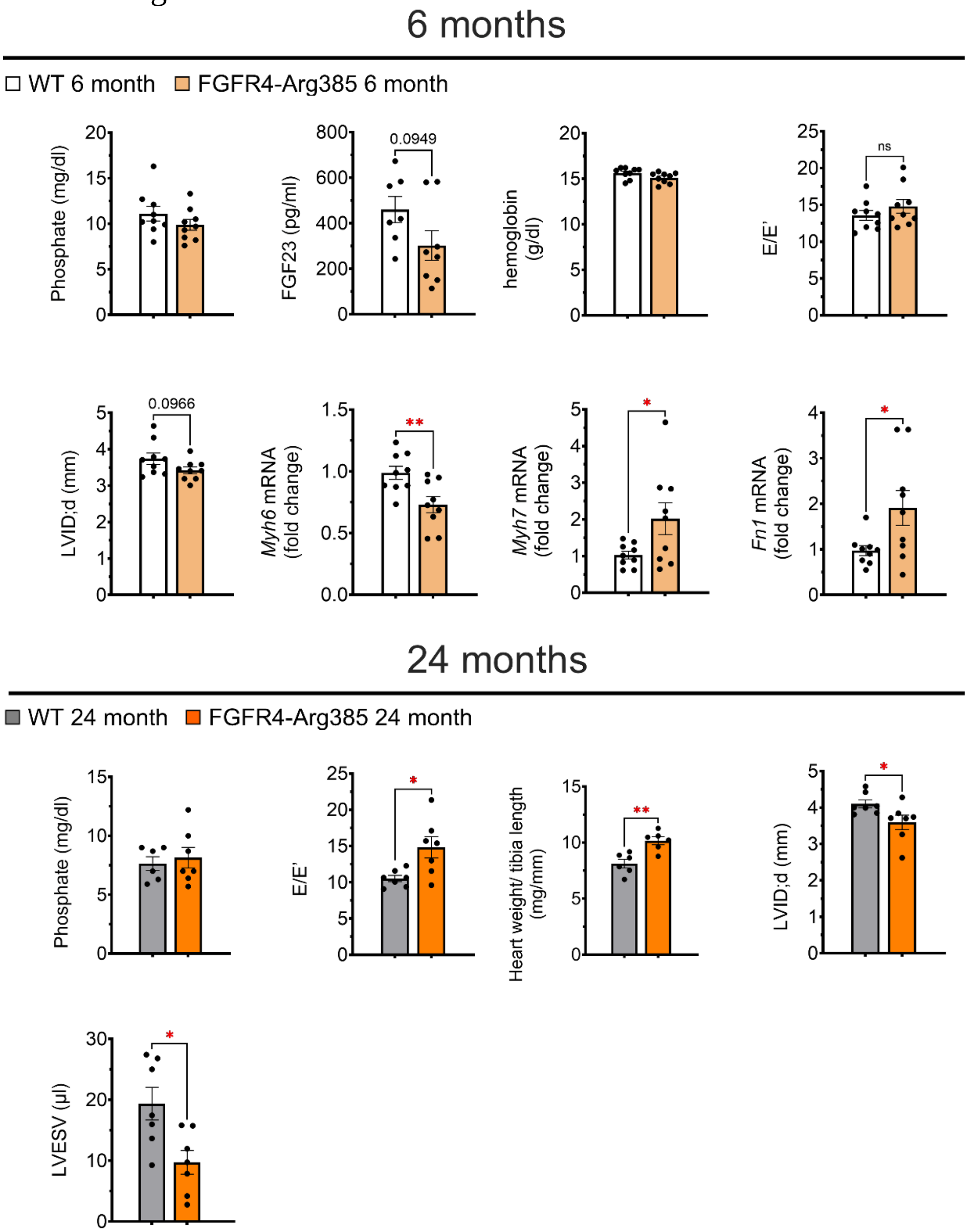

At 6 month of age, FGFR4-Arg385 mice showed no impaired cardiac or renal function. Expression of *Myh7* and *Fn1* mRNA however was already increased, indicating initiation of structural remodeling, but no detectable structural changes (top). Measurement of serum phosphate in

FGFR4-Arg385 mice at 24 month of age indicates no changes to mineral metabolism or renal function, but significant impairment of cardiac function, consistent with LVH/HFpEF (bottom).

Bar graphs represent mean ± SEM and individual values included in the graph. n ≥ 6 for all experiments. * $P < 0.05$; ** $P < 0.005$.

Supplemental Figure S5 – Renal and cardiac function in FGFR4$^{-/-}$ mice with CKD.

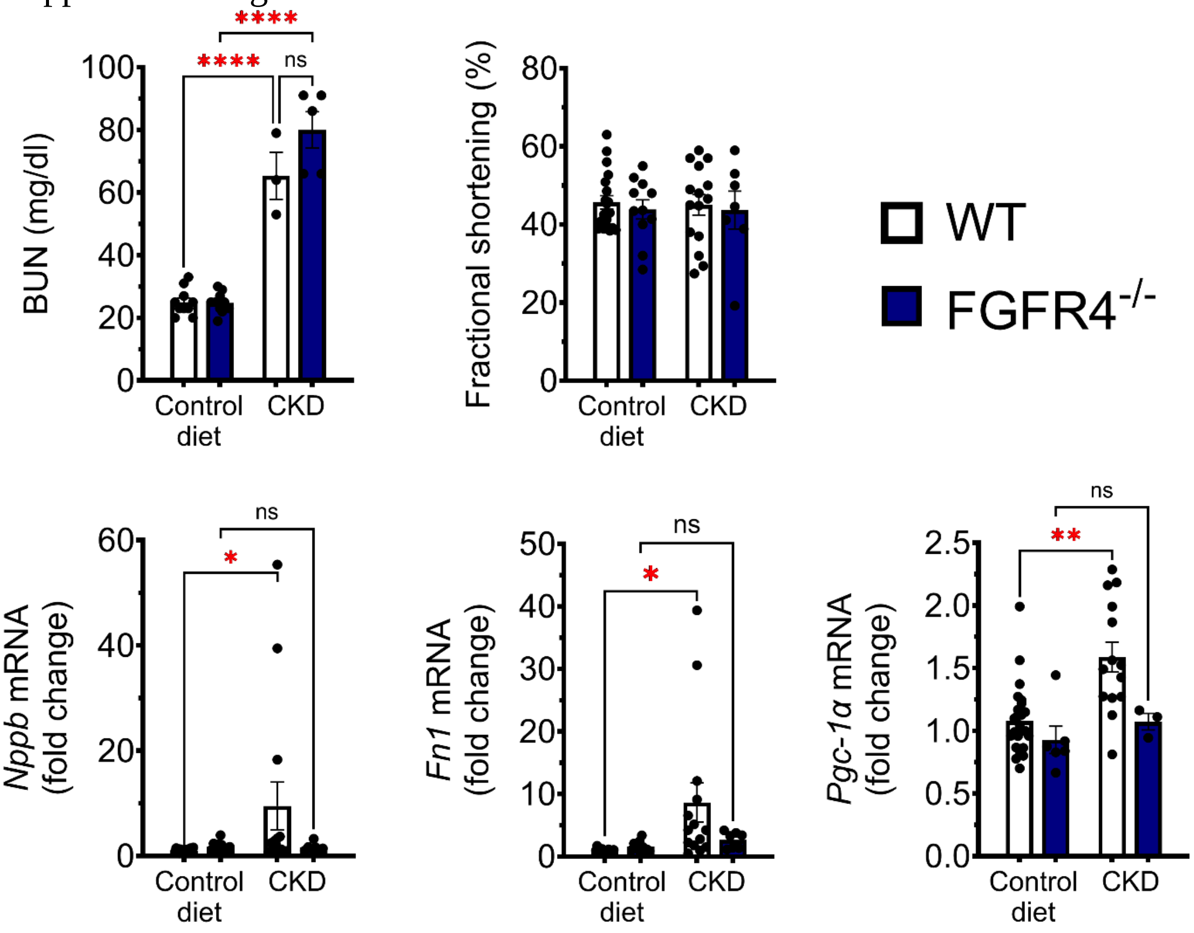

Measurement of BUN after 16 weeks on the adenine diet indicated FGFR4$^{-/-}$ mice develop CKD to the same degree as the control animals. Cardiac function, evaluated by fractional shortening was not changed between groups. Deletion of FGFR4 also normalized expression of cardiac pro-hypertrophic and pro-fibrotic markers.

Bar graphs represent mean ± SEM and individual values included in the graph. n ≥ 3 for all experiments. * $P < 0.05$; ** $P < 0.005$; **** $P < 0.0001$.

Supplemental Figure S6 - Tissue specific deletion of FGFR4 in cardiomyocytes and functional parameters.

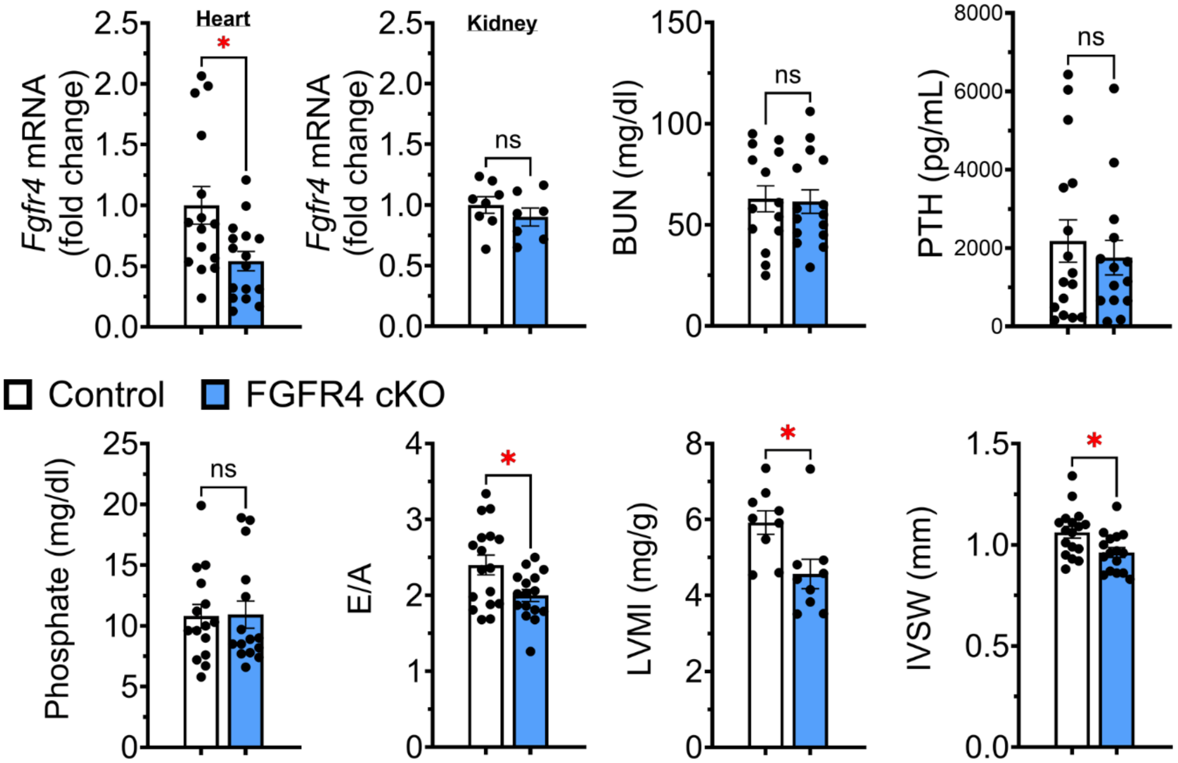

Deletion of FGFR4 under the α-MHC promotor was induced with a total of 3 injection of 30 mg/kg bodyweight tamoxifen, i.p. every 48h. Expression of *Fgfr4* in cardiomyocytes of α-MHC^{MerCreMer}-FGFR4^{flox} mice was measured 10 days after the last injection by qPCR. Abundance of kidney *Fgfr4* mRNA was measured to verify tissue specificity of the deletion. Serum levels of BUN, PTH and phosphate indicate the same degree of renal damage after 16 weeks on the adenine diet in control animals and α-MHC^{MerCreMer}-FGFR4^{flox} mice. Control, but not α-MHC^{MerCreMer}-FGFR4^{flox} mice, developed diastolic dysfunction marked by changes in E/A ratio with preserved systolic function.

Bar graphs represent mean ± SEM and individual values included in the graph. n ≥ 8 for all experiments. * P < 0.05.

Supplemental References

1. Hansen A, Eder A, Bönstrup M, et al. Development of a drug screening platform based on engineered heart tissue. *Circ Res*. 2010;107(1):35-44. doi:10.1161/CIRCRESAHA.109.211458

2. Breckwoldt K, Letuffe-Brenière D, Mannhardt I, et al. Differentiation of cardiomyocytes and generation of human engineered heart tissue. *Nat Protoc*. 2017;12(6):1177-1197. doi:10.1038/nprot.2017.033

3. Mannhardt I, Breckwoldt K, Letuffe-Brenière D, et al. human engineered heart tissue: analysis of contractile force. *Stem Cell Reports*. 2016;7(1):29-42. doi:10.1016/j.stemcr.2016.04.011

4. Helfer A, Bursac N. Frame-hydrogel methodology for engineering highly functional cardiac tissue constructs. *Methods Mol Biol*. 2021;2158:171-186. doi:10.1007/978-1-0716-0668-1_13

5. Sánchez-Aguilera P, López-Crisosto C, Norambuena-Soto I, et al. IGF-1 boosts mitochondrial function by a Ca2+ uptake-dependent mechanism in cultured human and rat cardiomyocytes. *Front Physiol*. 2023;14:1106662. doi:10.3389/fphys.2023.1106662

6. Rieg T. A High-throughput method for measurement of glomerular filtration rate in conscious mice. *J Vis Exp*. 2013;(75). doi:10.3791/50330

7. Ellery SJ, Cai X, Walker DD, et al. Transcutaneous measurement of glomerular filtration rate in small rodents: Through the skin for the win? *Nephrology*. 2015;20(3):117-123. doi:10.1111/nep.12363

8. Jordan CZ, Chen Y, Husain I, et al. Murine kidney transplant outcome is best measured by transdermal glomerular filtration rate. *American Journal of Transplantation*. 2024;24(12):2150-2156. doi:10.1016/j.ajt.2024.07.010

9. Abraham D, Mao L. Cardiac pressure-volume loop analysis using conductance catheters in mice. *J Vis Exp*. 2015;(103):52942. doi:10.3791/52942

10. Babraham Bioinformatics - Trim Galore! Accessed November 16, 2023. http://www.bioinformatics.babraham.ac.uk/projects/trim_galore/

11. Martin M. Cutadapt removes adapter sequences from high-throughput sequencing reads. *EMBnet.journal*. 2011;17(1):10-12. doi:10.14806/ej.17.1.200

12. Kersey PJ, Staines DM, Lawson D, et al. Ensembl Genomes: an integrative resource for genome-scale data from non-vertebrate species. *Nucleic Acids Research*. 2012;40(D1):D91-D97. doi:10.1093/nar/gkr895

13. Dobin A, Davis CA, Schlesinger F, et al. STAR: ultrafast universal RNA-seq aligner. *Bioinformatics*. 2013;29(1):15-21. doi:10.1093/bioinformatics/bts635

14. Anders S, Pyl PT, Huber W. HTSeq—a Python framework to work with high-throughput sequencing data. *Bioinformatics*. 2015;31(2):166-169. doi:10.1093/bioinformatics/btu638

15. Love MI, Huber W, Anders S. Moderated estimation of fold change and dispersion for RNA-seq data with DESeq2. *Genome Biology*. 2014;15(12):550. doi:10.1186/s13059-014-0550-8

16. Huber W, Carey VJ, Gentleman R, et al. Orchestrating high-throughput genomic analysis with Bioconductor. *Nat Methods*. 2015;12(2):115-121. doi:10.1038/nmeth.3252

17. R: The R Project for Statistical Computing. Accessed November 16, 2023. https://www.r-project.org/

18. Mootha VK, Lindgren CM, Eriksson KF, et al. PGC-1alpha-responsive genes involved in oxidative phosphorylation are coordinately downregulated in human diabetes. *Nat Genet*. 2003;34(3):267-273. doi:10.1038/ng1180

19. Ferrara CT, Wang P, Neto EC, et al. Genetic networks of liver metabolism revealed by integration of metabolic and transcriptional profiling. *PLoS Genet*. 2008;4(3):e1000034. doi:10.1371/journal.pgen.1000034

20. Newgard CB, An J, Bain JR, et al. A branched-chain amino acid-related metabolic signature that differentiates obese and lean humans and contributes to insulin resistance. *Cell Metab*. 2009;9(4):311-326. doi:10.1016/j.cmet.2009.02.002

21. Jensen MV, Joseph JW, Ilkayeva O, et al. Compensatory responses to pyruvate carboxylase suppression in islet beta-cells. Preservation of glucose-stimulated insulin secretion. *J Biol Chem*. 2006;281(31):22342-22351. doi:10.1074/jbc.M604350200

22. Schneider CA, Rasband WS, Eliceiri KW. NIH Image to ImageJ: 25 years of image analysis. *Nat Methods*. 2012;9(7):671-675. doi:10.1038/nmeth.2089

23. Pang Z, Lu Y, Zhou G, et al. MetaboAnalyst 6.0: towards a unified platform for metabolomics data processing, analysis and interpretation. *Nucleic Acids Research*. 2024;52(W1):W398-W406. doi:10.1093/nar/gkae253

24. Fang Z, Liu X, Peltz G. GSEApy: a comprehensive package for performing gene set enrichment analysis in Python. *Bioinformatics*. 2023;39(1):btac757. doi:10.1093/bioinformatics/btac757

25. v0.11.2 (August 2021) — seaborn 0.13.2 documentation. Accessed December 16, 2024. https://seaborn.pydata.org/whatsnew/v0.11.2.html
